# Consecutive seeding and transfer of genetic diversity in metastasis

**DOI:** 10.1073/pnas.1819408116

**Published:** 2019-06-25

**Authors:** Alexander Heyde, Johannes G. Reiter, Kamila Naxerova, Martin A. Nowak

**Affiliations:** ^a^Program for Evolutionary Dynamics, Harvard University, Cambridge, MA 02138;; ^b^Department of Organismic and Evolutionary Biology, Harvard University, Cambridge, MA 02138;; ^c^Canary Center for Cancer Early Detection, Department of Radiology, Stanford University School of Medicine, Palo Alto, CA 94304;; ^d^Center for Systems Biology, Massachusetts General Hospital and Harvard Medical School, Boston, MA 02114;; ^e^Department of Radiology, Massachusetts General Hospital and Harvard Medical School, Boston, MA 02114;; ^f^Department of Mathematics, Harvard University, Cambridge, MA 02138

**Keywords:** metastasis, clonal diversity, tumor heterogeneity, branching process, population genetics

## Abstract

During metastasis, only a fraction of genetic diversity in a primary tumor is passed on to metastases. We calculate this fraction of transferred diversity as a function of the seeding rate between tumors. At one extreme, if a metastasis is seeded by a single cell, then it inherits only the somatic mutations present in the founding cell, so that none of the diversity in the primary tumor is transmitted to the metastasis. In contrast, if a metastasis is seeded by multiple cells, then some genetic diversity in the primary tumor can be transmitted. We study a multitype branching process of metastasis growth that originates from a single cell but over time receives additional cells. We derive a surprisingly simple formula that relates the expected diversity of a metastasis to the diversity in the pool of seeding cells. We calculate the probability that a metastasis is polyclonal. We apply our framework to published datasets for which polyclonality has been previously reported, analyzing 68 ovarian cancer samples, 31 breast cancer samples, and 8 colorectal cancer samples from 15 patients. For these clonally diverse metastases, under typical metastasis growth conditions, we find that 10 to 150 cells seeded each metastasis and left surviving lineages between initial formation and clinical detection.

## Significance

The success of cancer treatment largely depends on the genetic mutations present within metastases, which cause 90% of cancer-related deaths. Genetically diverse metastases are more likely to harbor resistance mutations, contributing to treatment failure. It is often assumed that each metastasis is seeded exactly once, such that its diversity cannot be inherited and instead must emerge entirely during growth, yet many metastases have a diversity pattern inconsistent with this assumption. We introduce a mathematical model of consecutive seeding by multiple cells that can explain these patterns. We then apply this model to tumor sequencing data to infer that 10 to 150 cells seeded each metastasis. We derive predictions for the fraction of transferred diversity and the proportion of polyclonal lesions.

Intratumoral heterogeneity is an inevitable consequence of cancer evolution (1, 2). At the time of cancer diagnosis, many clones (subpopulations of genetically similar cells that share a common ancestry) coexist in the primary tumor (3, 4). When some of these clones give rise to metastases, the clonal heterogeneity present in the primary tumor is distributed to distant sites (5–8). Across cancer types, the mutations with the greatest predicted functional consequences are predominantly shared across all metastases, suggesting that these mutations first arose in the primary tumor and were then distributed to each metastasis (9). Since primary tumors are often surgically removed, the heterogeneity within metastases determines the probability for treatment efficacy (2, 3, 10).

While it frequently has been assumed that individual metastases are seeded exactly once by a single cell or a small cluster of similar cells (11, 12), recent studies have identified metastases with multiple subpopulations derived from different clones in the primary tumor (13–19). This transfer of clonal diversity suggests at least one of two possible mechanisms: that metastases can be seeded multiple times by different migrating cells (consecutive seeding) or that metastases can be seeded by a cluster of multiple clonally diverse cells (polyclonal cluster seeding). Although some empirical and theoretical work has suggested that circulating tumor cell clusters can be genetically diverse (20–22), the diversity established by polyclonal cluster seeding cannot necessarily be maintained during metastasis growth without consecutive seeding, as only a small number of cell lineages typically survive the stochastic growth process (17). However, established tumors may be consecutively seeded by an influx of cells from other tumors (23, 24), which presents a plausible mechanism for the dynamic transfer of clonal diversity between tumors.

**Fig. 1.**
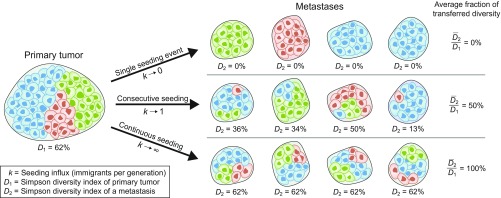
Seeding influx determines the intratumoral heterogeneity of metastases. A mature primary tumor, pictured at *Left* with N=3 clones (colored blue, green, or red) and a Simpson diversity index $D_{1}=62\%$, seeds M=4 metastases, each with a Simpson diversity index $D_{2}$. The average fraction of neutral clonal diversity in the primary tumor that is transferred to each metastasis depends only on the seeding influx k, defined as the mean number of cells that disseminate from the primary tumor to each metastasis per cell generation time. If k is very small (*Top* row), each metastasis is seeded only once by the primary tumor and hence will contain only one clone and no diversity. At the other extreme, if k is very large (*Bottom* row), each metastasis grows via continuous seeding from the primary tumor and hence will share the same genetic diversity as the primary tumor. We find that for all intermediate cases (*Middle* row), the average fraction of transferred neutral clonal diversity is ${\bar{D}}_{2}/D_{1}=k/\left(1+k\right)$.

The probability to successfully colonize a distant site depends on many factors (e.g., cancer type, metastatic potential, distance to site, and anatomy), described by the classical “seed and soil” hypothesis put forth more than a century ago (25–27). A consequence of this hypothesis is that if a primary tumor disseminates highly potent seeds to a perfectly compatible and nearby soil, this site will receive a constant stream of incoming and proliferating cancer cells. In contrast, a distant and unfavorable colonized site might receive one or very few cancer cells that can then expand. The seeding of metastases is therefore bounded by two extreme hypothetical scenarios: (*i*) A site is colonized by a single founding cell that expands by cell division to a detectable metastasis, such that the primary tumor and metastasis share only the mutations present in that founding cell, and (*ii*) a site is colonized by continuous influx of cancer cells and expands solely by this continuous influx, such that the primary tumor and metastasis on average contain the same genetic diversity (Fig. 1).

Many metastases might be established by a process which lies between these two extreme points, in which a tumor expands due to a balance of consecutive seeding events and subsequent cell divisions. However, previous mathematical models of metastasis have focused almost exclusively on extreme *i*, single-cell seeding (28–32). Although not yet studied in the context of cancer genetics, some mathematical models of consecutive immigration have been applied to other biological systems, in particular island populations (33–36). Yet these models from population genetics typically assume populations of fixed size (37), whereas the rapid growth of tumors can lead to dramatically different predictions (38, 39).

Here we develop a mathematical framework that generalizes past models to allow for multiple consecutive seeding events during tumor expansion, enabling us to assess and estimate the balance between seeding and cell division during the growth of metastases. This framework establishes a precise, quantitative mapping between the rates of seeding influx to each metastasis and the clonal diversity of metastases. This mapping can be used to predict tumor clonal diversity when information about the rate of seeding is known, and inversely it can be used to estimate the seeding rate from clone frequency data measured across multiple tumors within a patient.

## Model Formulation

We developed a mathematical framework using a multitype continuous-time branching process (4, 9, 40–42) to assess the dependence of metastatic heterogeneity on the seeding rate, birth rate, and death rate of cancer cells in a growing lesion (*SI Appendix*, Fig. S1). We consider a primary tumor that seeds M growing metastases and assess the composition of each metastasis once it has reached a detectable size Y. Each cell in the metastases derives from one of N clones, where every clone has a constant size in the primary tumor. Cells from each clone i=1,…,N arrive at a metastasis site with a constant seeding rate $\lambda_{i}$. This seeding rate of each clone reflects the product of three factors: the frequency of the clone in the primary tumor, the total size of the primary tumor, and the average likelihood of a cell in the clone to disseminate to the secondary site. This dissemination likelihood may depend on several additional factors, including the metastatic potential of a clone and the spatial arrangement of clones in the primary tumor.

After arriving at the new site, the cells from each clone i replicate according to an exponential birth–death process with division rate $b_{i}$ and death rate $d_{i}$, where $b_{i}>d_{i}$ (43, 44). Rather than characterizing each clone by its rates $\left(\lambda_{i},b_{i},d_{i}\right)$, our results take on a simpler form when expressed in terms of three related parameters, $\left(k_{i},\rho_{i},r_{i}\right)$. These parameters are the average influx of clone i cells per generation $k_{i}=\lambda_{i}/b_{i}$, the probability that a clone i seeding event establishes a surviving cell lineage $\rho_{i}=1-d_{i}/b_{i}$, and the average net growth rate $r_{i}=b_{i}-d_{i}$ of each clone i. The total seeding influx across all clone types is denoted as $k=\sum_{i=1}^{N}k_{i}$. We note that if time is measured in scaled units of average cell division time such that $b_{i}=1$, then simply $\rho_{i}=r_{i}$ and $k_{i}=\lambda_{i}$. For simplicity, we focus here on the case of neutral diversity in metastases (45–48). In this regime, all clones i share the same birth rate $b_{i}=b$ and death rate $d_{i}=d$ within a tumor, although these rates can freely vary between the tumors without affecting our predictions. For this neutral case, $\rho_{i}=\rho$ and $r_{i}=r$ are the same for all clones i in a tumor, but the seeding influxes $k_{i}$ can vary widely between clones. Results for the more general case of driver diversity (*SI Appendix*, Fig. S2) are reported in *SI Appendix*.

**Fig. 2.**
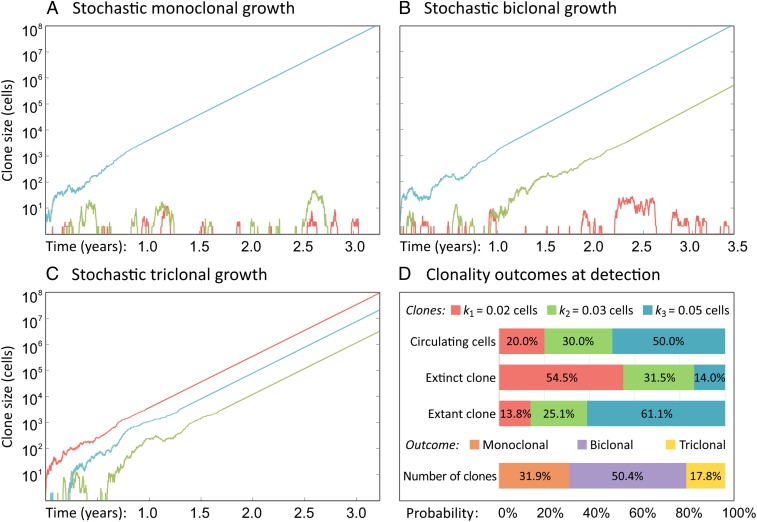
Stochasticity in metastasis growth leads to variable clonality outcomes. (*A*–*C*) Three sample realizations of metastasis growth to a detectable size $Y=10^{8}$ cells with growth rate r= 0.0125/d and survival probability ρ= 5%, as seeded by a primary tumor composed of N= 3 clones with seeding influxes $k_{1}=$ 0.02 (red), $k_{2}=$ 0.03 (green), and $k_{3}=$ 0.05 (blue) cells. Each panel depicts one of three potential outcomes—monoclonality, biclonality, and triclonality. (*D*) Our model leads to simple analytical results for (*i*) the average frequency of each clone in the circulating cells; (*ii*) the probability that each clone is extinct in a biclonal metastasis; (*iii*) the probability that each clone is extant in a monoclonal metastasis; and (*iv*) the relative likelihood of monoclonality, biclonality, and triclonality, i.e., the probability that there exist n= 1, 2, or 3 clones with nonzero frequency in a detected metastasis.

We evaluate the heterogeneity of a metastasis at a detection time T, defined as the first time that the total size of the metastasis $y_{i}\left(t\right)$ reaches the detection size Y. Simulated realizations with realistic parameter values (Table 1) highlight the diversity of possible metastases that can arise from the same primary tumor due to stochastic effects alone, even if all metastases share the same seeding and growth rates (Fig. 2). The same choice of parameter values can result in both monoclonal [i.e., a single clone is present in the evaluated metastasis (13, 50)] and polyclonal (i.e., more than one clone is present) metastases, underscoring the importance of stochastic effects in establishing clonal diversity.

**Table 1.** Model parameters and typical values

	Parameter	Typical values
r	Net metastasis growth rate	0.0125/day (39, 46)
ρ	Lineage survival probability	5.0% (48)
λ	Seeding rate to metastases	0.15–2.9 cells/day (28)
Y	Metastasis detection size	$10^{7}\text{–}10^{9}$ cells (49)
b	Cell division rate in metastasis	0.2500/d, r/ρ
d	Cell death rate in metastasis	0.2375/d, $r\left(\rho^{-1}-1\right)$
k	Mean cell influx per generation	0.6–11.6 cells, MLE
X	Total seeded surviving cells	10–150 cells, MLE

## Results

Our mathematical framework gives rise to several predictions about the shared genetic diversity, the proportion of polyclonal metastases, and the distribution of detection times for metastases growing with consecutive seeding. First, a key prediction of consecutive seeding is that the number of clones shared between the primary tumor and metastasis can increase over time as the metastasis grows and is consecutively seeded by cells from the primary tumor; this is a distinguishing feature from polyclonal cluster seeding, where the number of clones shared between the primary tumor and the metastasis decreases over time as lineages are lost to extinction (*SI Appendix*, Fig. S9). To investigate the clonal dynamics of metastasis growth under our model of consecutive seeding, we calculate the average size ${ȳ}_{i}\left(t\right)$ of each clone at time t by solving the equation ${ȳ}_{i}^{\prime }\left(t\right)=r{ȳ}_{i}\left(t\right)+\lambda_{i}$ that describes the expected growth and seeding dynamics, yielding

$${ȳ}_{i}\left(t\right)=\frac{k_{i}}{\rho }\left(e^{rt}-1\right)$$ [1]

which grows exponentially with rate r in the long run.

Because stochasticity in metastasis growth can lead to deviation from this mean behavior, we also computed the full probability distribution for the clone size $y_{i}\left(t\right)$ (*SI Appendix*). We find that the stochastic size $y_{i}\left(t\right)$ of each clone at time t follows a negative binomial distribution with two parameters,

$$y_{i}\left(t\right)∼\text{NBin}\left(k_{i},q_{i}\left(t\right)=\frac{k_{i}}{{ȳ}_{i}\left(t\right)+k_{i}}\right)$$ [2]

consistent with previous models involving stochastic population processes (51, 52). Two equivalent interpretations of this result provide complementary intuitions. First, $y_{i}\left(t\right)$ is equivalent to the number of successes before $k_{i}$ failures, each with failure probability $q_{i}\left(t\right)$, where for neutral diversity $q_{i}\left(t\right)$ is the same function for every clone i. Here “failure” refers to the event that a cell in the growing metastasis arrives from the primary tumor rather than being produced via cell division in the metastasis; this balance between seeding and birth rates is captured by the influx ratio $k_{i}=\lambda_{i}/b$. Second, following the lineage structure of clones in the metastasis, $y_{i}\left(t\right)$ can be interpreted as the number of cells in each surviving lineage at time t, summed over all surviving lineages. We analyze this alternative construction by deriving the number of distinct cell lineages and their respective sizes in *SI Appendix*.

To assess the clonal composition of a detected metastasis, we define $Y_{i}$ to be the number of cells in a metastasis of size Y descended from the ith clone in the primary tumor, so that $Y=\sum_{i=1}^{N}Y_{i}$. We show that the detected clone sizes jointly follow a Dirichlet-multinomial distribution,

P(Y1,…,YN)=∏i=1NYi+ki−1YiY+k−1Y [3]

which, following the derivation in *SI Appendix*, emerges from the Pólya urn scheme of sampling with double replacement. In this statistical scheme, the clonal membership of each cell in a metastasis is evaluated in sequence: For the first cell, sampled at random, its probability to be of a particular clone is simply given by the prior distribution of clone sizes in the primary tumor; but once the clonal membership of the first cell is identified, the probability that the second cell is of the same clone is increased relative to the prior distribution, and so on for each cell identified in this manner.

This scheme can be applied to evaluate the number of clones n present with nonzero size in a metastasis of size Y. We find that the mean number of clones n present in the metastasis is

n¯=N−∑i=1NY+k−ki−1YY+k−1Y [4]

and the probability that a metastasis is polyclonal (composed of multiple clones with nonzero size) or, equivalently, the expected fraction of polyclonal metastases in a patient, is

**Fig. 3.**
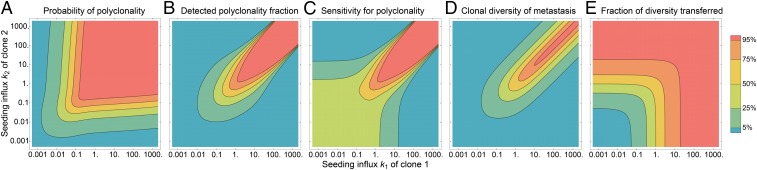
Metastasis clonality and clonal diversity vary with seeding influx. Each contour plot visualizes analytical results for a metastasis of size $Y=10^{8}$ cells seeded by a primary tumor with two neutral clones N=2, each with seeding influxes $k_{1}$ and $k_{2}$. (*A*) The probability that a detected metastasis is polyclonal is greatest when the clones have high but balanced seeding rates. (*B*) The probability that a detected metastasis is polyclonal is calculated using a minimum cell fraction threshold of 5% for each clone. If the total seeding influx $k=k_{1}+k_{2}$ is high and the ratio of influxes is of order $k_{1}/k_{2}∼10^{\pm 2}$, then the tumor is likely to have undetected polyclonality. (*C*) The sensitivity for polyclonality, defined as the mean fraction of polyclonal metastases that are detected as polyclonal (i.e., the ratio of *B* to *A*), is lowest when the clones have very different seeding rates. (*D*) The expected clonal diversity of a detected metastasis is calculated as twice the probability that two cells chosen at random from the metastasis are descendants of different clones (Simpson diversity index). Metastases are most clonally diverse when they are also most likely to be identified as polyclonal. (*E*) The mean fraction of clonal diversity present in the primary tumor that is transferred to a metastasis depends only on the total relative seeding rate $k=k_{1}+k_{2}$ according to Eq. **7**.

P(n>1)=1−∑i=1NY+ki−1YY+k−1Y. [5]

(Fig. 3*A* and *SI Appendix*). This polyclonality probability is greatest when multiple clones have a high seeding influx. If only one clone has a high influx, or if all clones have a low influx, then polyclonality will be rarely detected because one clone dominates the metastasis (Fig. 3 *B* and *C* and *SI Appendix*, Fig. S3 *A* and *B*). In the particular case that each clone has an equal and small seeding rate k≪N, the probability of polyclonality is very well approximated by the simpler expression $P\left(n>1\right)\approx 1-\kappa !Y^{-\kappa },$ where κ=k(1−1/N) is the clone-adjusted influx; this probability increases with the seeding influx k per generation, the number of clones N in the primary tumor, and the total size Y of the metastasis. Here, monoclonality is more likely than polyclonality if the seeding rate is low, $\kappa <{\left(\log_{2}Y\right)}^{-1}$, or if the metastasis size is small, $Y<2^{1/\kappa }$; in contrast, polyclonality is more likely if the reverse is true.

In practice, clones and their population sizes are not measured directly and are instead approximated using mutation frequencies in bulk sequencing samples (4, 53). We therefore adapt our results, denoting the frequency of each clone i in the metastasis as $\gamma_{i}=Y_{i}/Y$. The mean clone frequencies are then simply the fraction of migrants that are of clone type i, such that ${\bar{\gamma }}_{i}=k_{i}/k$. In *SI Appendix*, we show that the vector of clone frequencies $\left(\gamma_{i},\ldots ,\gamma_{N}\right)$ follows a Dirichlet distribution,

$$P\left(\gamma_{1},\ldots ,\gamma_{N}\right)=\Gamma \left(k\right)\overset{N}{\underset{i=1}{\prod }}\frac{\gamma_{i}^{k_{i}-1}}{\Gamma \left(k_{i}\right)}.$$ [6]

The Dirichlet distribution is the multivariate generalization of the Balding–Nichols distribution that is widely used in the forensic analysis of genetic profiles (54). This result provides a remarkably clean and simple way to predict the complete distribution of clone frequencies within a metastasis given the seeding influx parameters of each clone. Moreover, this implies that for a single clone or mutation of interest with frequency ${\bar{\gamma }}_{i}$ in the primary tumor, the corresponding frequency $\gamma_{i}$ in a metastasis will marginally follow the Beta distribution, $\gamma_{i}∼\text{Beta}\left(k{\bar{\gamma }}_{i},k\left(1-{\bar{\gamma }}_{i}\right)\right)$, with a variance ${\bar{\gamma }}_{i}\left(1-{\bar{\gamma }}_{i}\right)/\left(1+k\right)$ that varies inversely with the total seeding rate k.

This precise mapping between the clonal composition of the primary tumor and its metastases, mediated by the seeding rates, can be simplified when considering only the clonal diversity of the tumors, rather than the full set of clone frequencies. Clonal diversity, measured on a scale 0 (least diverse) to 1 (most diverse), is a simple but informative summary metric for clonal composition; a natural measure of the clonal diversity of a tumor is the Simpson index, defined here as the probability that two cells selected at random from the metastasis are heteroclonal (descendants from different clones) (55). In a large tumor, this is calculated according to the expression $D=1-\sum_{i=1}^{N}\gamma_{i}^{2}$. For example, if n clones were present at equal frequencies, then the clonal diversity would be D=1−1/n. Inversely, given the mean clonal diversity *D* of a tumor, the fraction 1/(1−D) provides a rough estimate for the “effective” number of clones in a tumor in which all clones were equally abundant. When the clonal diversity of the primary tumor is high, the average clonal diversity of a metastasis will be similarly high if and only if the total seeding influx k is much greater than unity (Fig. 3*D*).

Moreover, in our analytic framework, the ratio of the mean clonal diversity ${\bar{D}}_{2}$ of a metastasis to the clonal diversity $D_{1}$ of the primary tumor that seeded it is a simple function of the seeding influx k between the tumors,

$$\frac{{\bar{D}}_{2}}{D_{1}}=\frac{k}{1+k}=\frac{\lambda }{b+\lambda }$$ [7]

(Fig. 3*E* and *SI Appendix*, Fig. S3*E*). This ratio can be interpreted as the mean fraction of clonal diversity that is disseminated from the primary tumor to the metastasis. This analysis can also be extended to quantify intermetastatic heterogeneity (2, 9): If a primary tumor seeds M metastases with equal rates, the difference in clone composition among the metastases is captured by the fixation index $F_{ST}$. In our framework,

$$F_{ST}=1-\frac{{\bar{D}}_{2}}{D_{2}^{*}}=\left(1-\frac{1}{M}\right)\frac{1}{1+k},$$ [8]

where $D_{2}^{*}$ denotes the mean clonal diversity over the aggregate population of cells across all metastases (*SI Appendix*, Fig. S3*F*). This quantity, a standard measure of clonal differentiation in population genetics (54, 56, 57), can be readily estimated from genetic data collected from spatially segregated metastases (58, 59). From the above expression, we find that as additional metastases are seeded, the clonal diversity of the aggregate metastatic population will converge to that of the primary tumor, $D_{2}^{*}\to D_{1}$, and so $F_{ST}\to 1/\left(1+k\right)$ for large M.

Because the above results make predictions about clonal diversity given the seeding influxes of each clone, we can invert our model to infer the seeding influxes from measurements of clonal frequencies across multiple tumors in a patient. In this inference approach, we observe the clonal frequencies $\gamma_{ij}$ of each clone i in each tumor j, and we estimate the corresponding seeding influxes $k_{ij}={\bar{\gamma }}_{i}\cdot k_{j}$, where ${\bar{\gamma }}_{i}$ denotes the mean clone frequencies in the primary tumor and $k_{j}$ denotes the estimated total seeding influx to tumor j. Using maximum-likelihood estimation (MLE), we derive that these estimates should be chosen to jointly satisfy the conditions $\Sigma_{j=1}^{N}k_{j}\cdot \beta_{ij}=0$ for all clones i and $\Sigma_{i=1}^{N}{\bar{\gamma }}_{i}\cdot \beta_{ij}=0$ for all tumors j, where $\beta_{ij}=\ln\left(\gamma_{ij}\right)-\left[\psi \left(k_{ij}\right)-\psi \left(k_{j}\right)\right]$ is the sample bias in the log-scaled clone frequencies (*SI Appendix*). If the clonal composition of the primary tumor ${\bar{\gamma }}_{i}$ is already known for all clones i, then the latter condition alone allows for the independent estimation of the seeding influxes $k_{j}$ to all tumors j (*SI Appendix*, Fig. S4). To first order, the MLE seeding influx ${\hat{k}}_{j}$ scales inversely with the Kullback–Leibler (KL) divergence $D_{KL}\left(\bar{\gamma }\|\gamma_{j}\right)=\sum_{i=1}^{N}{\bar{\gamma }}_{i}\ln\left({\bar{\gamma }}_{i}/\gamma_{ij}\right)$ between the clonal composition of a metastasis and the primary tumor that seeded it (*SI Appendix*, Fig. S5*A*). Specifically, in *SI Appendix* we show that

$${\hat{k}}_{j}\approx \frac{N-1}{\alpha \;D_{KL}\left(\bar{\gamma }\|\gamma_{j}\right)},$$ [9]

where α is bounded by the two extremes α=1 in the regime of high KL divergence ($D_{KL}\gg N$) and α=2 in the regime of low KL divergence ($D_{KL}\ll \frac{N}{2}\min_{i}{\bar{\gamma }}_{i}$). This scaling law, a fast approximation for the MLE seeding influx, quantifies the inverse relationship between the amount of consecutive seeding between two tumors and the resulting divergence in their clonal compositions. The uncertainty $\sigma_{j}^{2}$ in the estimate $\ln{\hat{k}}_{j}$ scales inversely with the number of clones, $\sigma_{j}^{2}=\alpha /\left(N-1\right)$. Hence a 95% confidence interval for the seeding influx can be constructed by computing the bounds ${\hat{k}}_{j}e^{\pm 1.96\sigma_{j}}$, giving an upper and lower estimate for the seeding influx to each tumor.

To demonstrate how this model-based inference approach can be used, we identified three published studies that reported sequencing results from multiple tumors collected simultaneously from a patient and revealed a pattern of at least two shared clones between tumors (13–15). Because these patterns can be explained only by several cells seeding a tumor, rather than just one, these datasets were appropriate for our inference approach; any dataset consistent with a single-cell seeding model would result in a maximum-likelihood estimate of zero consecutive seeding in our framework. In cases where multiple samples from a patient were collected from the originating organ and the true primary tumor site was unclear in the literature (16), inference was conducted across all tumor samples jointly regardless of anatomical location.

**Fig. 4.**
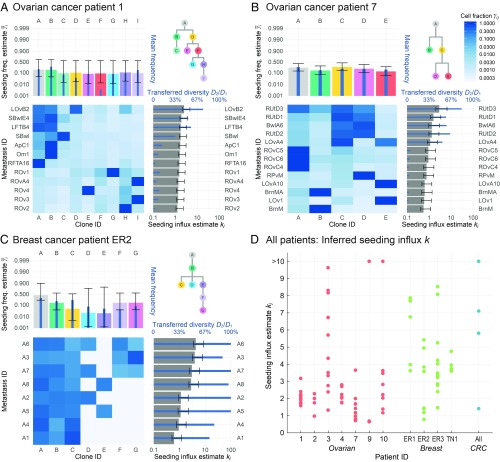
Inference of seeding influx from clonal frequency data collected from patients with ovarian cancer, breast cancer, and colorectal cancer (CRC). (*A–C*) In each panel, the heatmap shows the clonal composition of several sequenced tumor samples in a patient (colorkey at *Top Right*), as reported by McPherson *et al*. (13) and Savas *et al*. (14). As estimated by MLE over the distribution given by Eq. **6**, the seeding frequencies ${\bar{\gamma }}_{i}$ of each clone i are depicted as wide colored bars in the *Top Left* bar chart and the seeding influxes $k_{j}$ for each tumor j (the mean number of arriving cells per generation time) as wide gray bars in the *Bottom Right* bar chart, with black SE bars. In addition, the narrow blue bars depict the mean clone frequencies across all metastases (*Top Left* bar chart) and the fraction of diversity transferred from the circulating cells to each metastasis (*Bottom Right* bar chart). The tree in each panel (*Top Right*) depicts the inferred phylogenetic relationship of the detected clones in the patient. (*D*) Each solid circle, colored by cancer type, represents the estimated seeding influx $k_{j}$ for a single tumor sample. For nearly all samples included in this analysis, this estimate is consistently in the range 1 to 10 cells per generation.

First, using a clone frequency dataset from whole-genome sequencing of 68 tumor samples across 7 patients with high-grade serous ovarian cancer with intraperitoneal metastasis (13), we apply our MLE approach to estimate the seeding influx of each clone (Fig. 4 and *SI Appendix*, Fig. S6). Peritoneal metastasis represents an ideal test case for our inference approach because cancer cells that enter the peritoneal cavity are thought to mix easily within this space, facilitating consecutive seeding. We find that our total seeding influx estimates span the range $0.6<k_{j}<11.6$ cells per generation per tumor for all 68 tumor samples, with a mean of 2.7 cells across all patients. These estimates suggest that the average metastasis of a patient with ovarian cancer will be seeded by several cells during its growth and even several cells per generation of growth. The wide range of these estimates is in part due to heterogeneity between patients; patients 3 and 10 for example had high estimated seeding influxes with means 5.1 and 3.6 cells, respectively, while patients 2 and 7 had slightly lower estimated seeding influxes with means 1.7 and 1.4 cells, respectively. The remainder of the variability is then due to heterogeneity in the seeding influx between the tumors of each patient. Because our jointly estimated clone seeding frequencies also vary across a broad range, $4\%<{\bar{\gamma }}_{i}<40\%$ (mean = 16%), the estimated per-clone seeding influxes span a wider range $0.06<k_{ij}<4.0$ cells than the total seeding influxes $k_{j}$, with a mean of 0.41 cells per generation per tumor per clone. This wide range of inferred influxes, spanning nearly two orders of magnitude, suggests that some clones may have had a substantial seeding advantage over other clones, due in part to unequal clone sizes in the primary tumor.

We analyzed a second dataset of 31 tumor samples from 4 patients with metastatic breast cancer (14), and surprisingly we find qualitatively similar results (Fig. 4 and *SI Appendix*, Fig. S7), although in these cases metastasis must have occurred through lymphatic or hematogenous routes. The total seeding influx estimates vary in the range $0.7<k_{j}<8.6$ cells, with a mean of 4.0 cells across all patients. This mean estimate is greater than the analogous mean estimate of 2.7 cells for the patients with ovarian cancer, reflecting the considerable clonal diversity of the 4 patients with breast cancer included in this dataset. In particular, patient ER1 had the highest estimated seeding influxes with a mean of 6.0 cells, while patient ER2 had the lowest mean of 1.8 cells. Across all breast cancer clones, the estimated per-clone seeding influxes again span a wide range, $0.04<k_{ij}<4.2$ cells, with a mean of 0.71 cells, pointing to substantial clonal variation in seeding potential. We note that these were autopsy samples with very advanced disease, in contrast to the patients with ovarian cancer, and that larger metastases are more likely to be clonally diverse under a model of consecutive seeding.

We also studied 4 pairs of primary tumors and metastases from patients with colorectal cancer (15) and again estimated similar seeding influx values. Patients A01, A02, and A04 had MLE seeding influxes of 1.4, 7.1, and 5.8 cells, respectively. However, patient A03 had a primary tumor and metastasis with very similar clonal compositions, leading to an unusually high MLE seeding estimate of 114.5 cells. We note that patient A03 had the smallest number of clones (n=3) of every patient we examined and only a single metastasis, providing the least usable information for our inference approach.

For every sample in our analysis, our 95% confidence interval for $k_{j}$ spans less than half an order of magnitude (∼3.1-fold) on either side of our estimate, with an average SE of 1.64-fold in the ovarian cohort, 1.68-fold in the breast cohort, and 1.71-fold in the colorectal cohort. We conclude that the true seeding influx is no more than half an order of magnitude separated from the inferred values we obtained by maximum-likelihood estimation. In addition, because the clone frequency data $\gamma_{ij}$ may be subject to measurement error, we tested the robustness of our inference approach (*Materials and Methods*). We find that even when we introduce substantial measurement error of more than 50%, our maximum-likelihood estimates are robust, changing by less than 6.3% (*SI Appendix*, Fig. S5*B*), indicating that our framework is robust even to large amounts of uncorrelated noise in the data.

Using the estimated k values from our inference results, we can infer the total number of cells X that migrated to each tumor before detection and gave rise to a surviving lineage according to the expression for its expected value,

$$\bar{X}=\rho \lambda \bar{T}=k\left[\ln\left(\rho Y\right)-\psi \left(k\right)\right],$$ [10]

where $\bar{T}$ is the average time until the metastasis reaches detection size Y (*SI Appendix*, Fig. S3 *C* and *D*). In the patients with ovarian cancer, for a typical survival probability of ρ=5.0% and a metastasis detection size of $Y=10^{8}$ cells (Table 1), in conjunction with our MLE values of k, we estimate that X=38.4 (±30.6 SD) cells arrived at each tumor and gave rise to a surviving lineage during its growth. For comparison, using a maximum-parsimony approach for the same cohort of patients with ovarian cancer, El-Kebir *et al*. (16) find that a minimum of 6 to 10 consecutive seeding events (or “comigrations”) per patient are necessary to explain the observed clone patterns across samples. Because our MLE value is chosen to correspond to the most likely number of cells, rather than the smallest possible (most parsimonious) number, our estimates consistently exceed this minimum, as expected.

In the patients with breast cancer, we estimate X=56.8 (±26.4) cells, and in the patients with colorectal cancer excluding patient A03, we estimate X=65.9 (±39.1) cells. These estimates were calculated using the same typical parameter values, although they do not depend on the net growth rate r, so we do not necessarily assume that all cancer types grow at the same rate. Across all samples, the minimum and maximum estimates were 10.6 and 151 cells. Estimates for each patient and clone are provided in *SI Appendix*, Table S1 and visualized in Fig. 4 and *SI Appendix*, Figs. S6 and S7. These estimates are more accurate when measurements of ρ and Y are known, as $\bar{X}$ increases logarithmically with the product of these parameters (*SI Appendix*, Fig. S3). In particular, for larger metastases with $Y=10^{9}$ cells, we obtain estimates between a minimum of 11.6 cells and a maximum of 178 cells, while for smaller metastases with $Y=10^{7}$ cells, we obtain a slightly lower range of 8.8 to 124 cells that seed surviving lineages.

## Discussion

The presented mathematical framework quantitatively captures the stochasticity of metastatic seeding, cell division, and cell death, as well as clonal competition during the colonization of distant sites. We have derived from this stochastic framework a set of baseline predictions for clonal diversity that can be readily compared with observations as a means of evaluating to what extent these simple principles can explain the observed range of clonal complexity. We demonstrate that continuous seeding, as a mechanism for the transfer of clonal diversity between tumors (13, 50), can act as a filter of intratumoral heterogeneity and thereby influence the probability of resistance and treatment success (3, 46, 60). Given measurements of only 3 independent parameters, the model predicts the number of clones that are transferred to each metastasis before its detection (Eq. **4**), the fraction of polyclonal metastases (Eq. **5**), the distribution of clone frequencies in each metastasis (Eq. **6**), the expected fraction of clonal diversity transferred to a metastasis (Eq. **7**), and several other quantities of interest.

These model predictions can be inverted to provide a means of estimating the seeding influxes and mean clone frequencies in the primary tumor. Our analysis of 68 tumor samples from patients with ovarian cancer, 31 tumor samples from patients with breast cancer, and 4 pairs of primary tumor and metastasis samples from patients with colorectal cancer yielded seeding influx estimates consistently in the range 0.6 to 11.6 cells per generation time. These datasets were chosen because they include explicitly reported clone frequencies. Our high seeding influx estimates reflect the high degree of shared clonal diversity observed in the patients included in these datasets, as it is likely that these patients have higher seeding influxes than most patients with cancer. We note that, in contrast to the suggestion of McPherson *et al*. (13), our model demonstrates that invoking a nonuniform fitness landscape is not required to explain the high proportion of polyclonal metastases observed in some patients with cancer. Rather, the stochastic features of metastasis growth, coupled with a seeding influx that falls in the range 0.6 to 11.6 cells per generation time, are sufficient to explain these observations.

The simple, analytical form of our results reveals how various quantities precisely depend on the model parameters and provides a means of calculating these quantities without the need for computationally expensive numerical simulation. As such, these results may be readily integrated in computational methods that seek to infer the clonal composition of tumors and their metastatic seeding patterns (4, 16, 50). We note several simplifying assumptions made to ensure tractability of the model. First, we assume that metastasis occurs after the primary tumor has reached a steady size and stable clonal composition. Consequently, the model may underestimate the variance in some predictions by neglecting possible fluctuations in the primary tumor size and clonal frequencies. In cases of early metastasis, these fluctuations have been modeled according to an upstream branching process in the primary tumor (9, 40). Very high seeding influxes k or survival probabilities ρ would increase the probability that surviving lineages are seeded early during primary tumor growth. Second, we model only the clonal diversity established in the primary tumor and not new clones that may arise in a growing metastasis. These new clones may be rare due to low mutation rates and relatively unlikely to outcompete established clones (9, 61, 62). Third, it is possible that the dissemination rate $\lambda_{i}$ and survival probability $\rho_{i}$ of newly seeded clones may not be constant as our model assumes, but instead vary with the size or clonal composition of the tumor, as could be the case if epistatic interactions between clones were significant. Finally, our seeding influx estimates are inferred from clone frequency data that may be subject to measurement noise and uncertainty (59), although we note that our estimates are quite robust if this noise is uncorrelated among clones (*SI Appendix*, Fig. S5*B*).

Our results describe properties of unidirectional consecutive seeding from a primary tumor to metastases and do not explicitly account for seeding between metastases (*SI Appendix*, Fig. S8). Nonetheless, our model can provide a useful approximation even in more complicated seeding scenarios. If a metastasis Z is seeded by another metastasis Y (with equal parameters governing the growth of both) rather than by the primary tumor X, the first seeding event on average occurs when metastasis Y is already a fraction 56%/k of its mature size (*SI Appendix*). Since at this size the clonal fractions in the tumor are stable, our inference framework for the seeding influx is not significantly affected. This result applies equally well to reseeding or self-seeding, in which cells that have left the primary tumor later return (23, 24), because metastasis Z can represent the population of the primary tumor X that has ancestry in metastasis Y. Then only k surviving cells return to the primary tumor during metastasis growth, again resulting in a negligible effect on neutral clone frequencies in either tumor (*SI Appendix*). Even when the reseeding outflux is as high as twice the seeding influx k, neglecting reseeding altogether has minimal effect on our seeding estimates (*SI Appendix*, Fig. S5*C*).

Intratumoral heterogeneity, a facilitator of treatment resistance and tumor relapse, is directly mediated by the seeding dynamics of cancer cells. Cancers characterized by a high rate of cell dissemination and mixing are especially likely to give rise to highly heterogeneous metastases as the cancer progresses. Our model of the transfer of clonal diversity between tumors, along with the corresponding analytical results and inference approach developed in this work, provides the tools to predict the genetic diversity and differentiation index of metastases, as well as to estimate the seeding influxes that gave rise to that diversity. Metastasis is a stochastic process that can generate considerable intratumoral heterogeneity, and understanding its role in determining this heterogeneity will be an important step toward providing more effective treatment.

## Materials and Methods

### Model.

We model the growth and evolution of a metastatic lesion as a continuous-time multitype branching process (40, 43, 44). Each lesion originates from a single cell but is consecutively seeded by additional cells over time. For more details, see *Model Formulation*.

### Analysis.

Using the mathematical properties of a Poisson process to describe consecutive seeding events, we derive several statistical quantities of interest in a stochastic setting. Full details and derivations of our results are provided in *SI Appendix*.

### Simulations.

We simulate the multitype branching process using the Gillespie algorithm (63) until a total tumor size of Y cells is achieved. For statistics, we conduct 100,000 independent realizations of our simulation for each set of model parameters (Table 1).

### Robustness.

For each of M=1,000 simulated tumor samples, we drew a true seeding influx k from a lognormal distribution and clone frequencies $\gamma_{ij}$ from Eq. **6**. After multiplying each frequency by an independent multiplicative error factor and renormalizing, we computed the MLE influx ${\hat{k}}_{\xi }$. See *SI Appendix* for further details.

### Patient Data.

All patient data analyzed in this study was previously published across 3 separate studies (13–15). In each study, tumor samples were collected with ethical approval by the institutional review board, and patients gave informed consent.

## Supplementary Material

Supplementary File
