# Supplementary material for: Consecutive seeding and transfer of genetic diversity in metastasis
Source: Proc Natl Acad Sci U S A. 2019 Jun 25;116(28):14129–37. doi: 10.1073/pnas.1819408116 (PMC6628640; doi:10.1073/pnas.1819408116)
Supplement: Supplementary File [file pnas.1819408116.sapp.pdf]

Supplementary Materials for

Consecutive seeding and reduction of clonal diversity in metastasis

Alexander Heyde, Johannes G. Reiter, Kamila Naxerova, Martin A. Nowak

Supplementary Figures

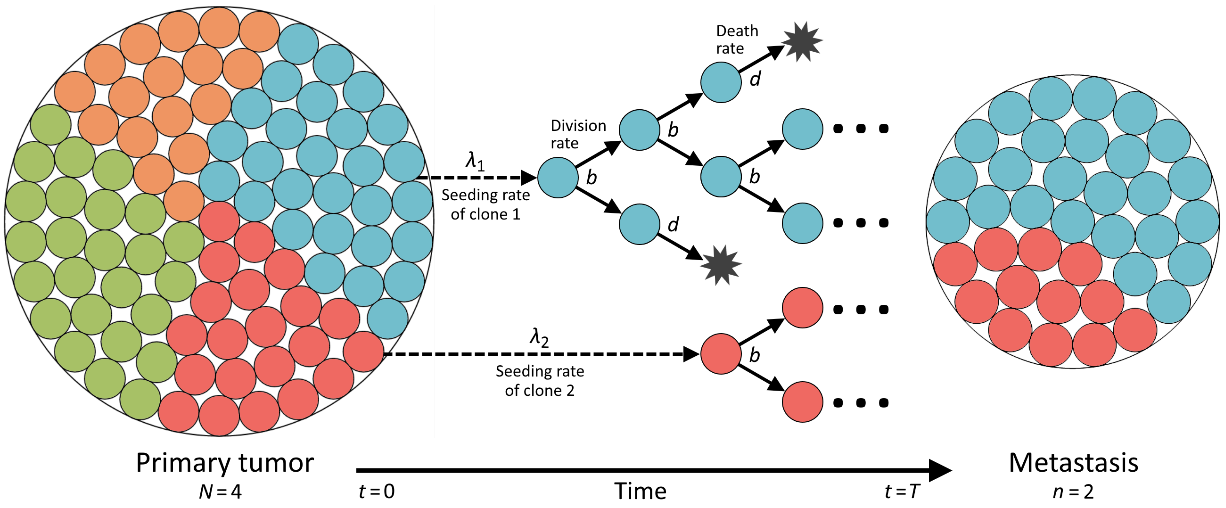

Supplementary Figure 1: Schematic of stochastic birth-death-seeding process. The mature primary tumor of fixed size and clonal diversity is composed of x_i cells of each clonal type $i = 1, \ldots, N$, represented by circles of different colors for the $N = 4$ clones pictured above. The growing metastasis is composed of $y_i(t)$ cells of each type i at time t. The metastasis is initialized with zero size at time $t = 0$ and is studied until it reaches a total detectable cell count Y at time $t = T$. Cells of each type i seed the metastasis at rate λ_i, where they then replicate at rate b and die at rate d. If the birth and death rates are set globally, this model can be used to analyze the dissemination of passenger diversity from a primary tumor to a metastasis, or the birth and death rates can vary between clones to capture selection in the presence of mosaic driver mutations.

www.pnas.org/cgi/doi/10.1073/pnas.1819408116

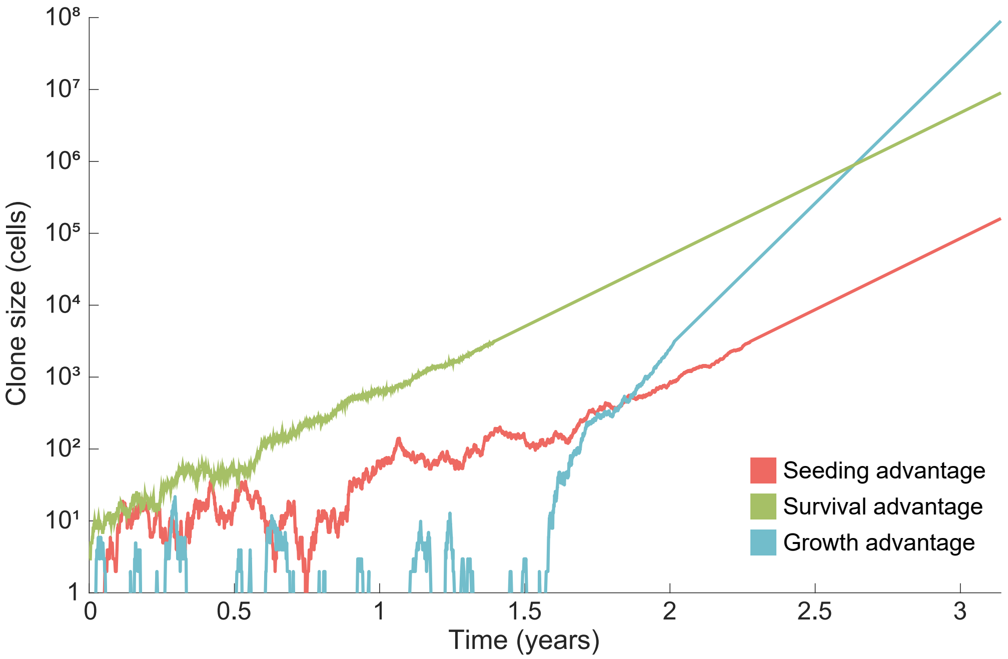

Supplementary Figure 2: Clonal competition with different driver advantages. A simulated realization of metastasis growth in which the primary tumor is composed of three subpopulations, each with a single driver advantage: a twofold greater seeding influx k (red), a twofold greater survival probability ρ (green), or a twofold greater net growth rate r (blue). The baseline parameters used are $k = 0.10$ cells, $r = 0.0125$ days^{-1}, and $\rho = 5.0\%$.

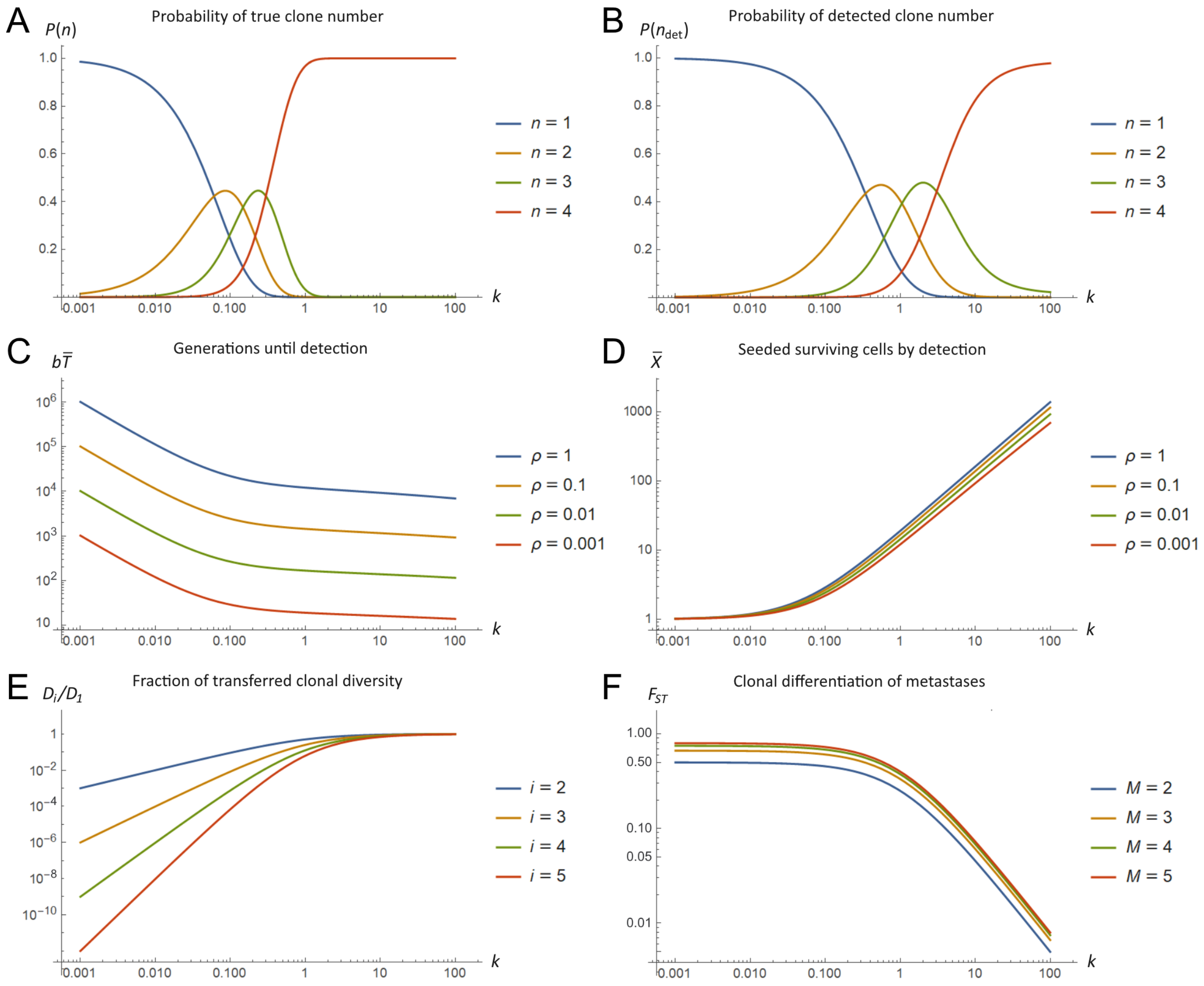

Supplementary Figure 3: Dependence of analytical results on the seeding influx. Various quantities are plotted as a function of the total seeding influx k for a metastasis of size $Y = 10^8$. (A) The probability distribution for the number of clones n at nonzero (>0%) frequency in the detected metastasis. (B) The probability distribution for the number of clones $n_{\rm det}$ at detectable (>5%) frequency in the detected metastasis. (C) The mean number of generations $b\bar{T}$ until the metastasis is detected, for various values of the survival probability ρ. (D) The average number of cells $\bar{X}$ seeded before detection that survive, for various values of the survival probability ρ. (E) The mean fraction of clonal diversity D_i/D_1 transferred from a primary tumor to an $i^{\rm th}$ order tumor after i sequential seeding steps. (F) The clonal differentiation F_{ST} of M metastases, each seeded directly by the primary tumor.

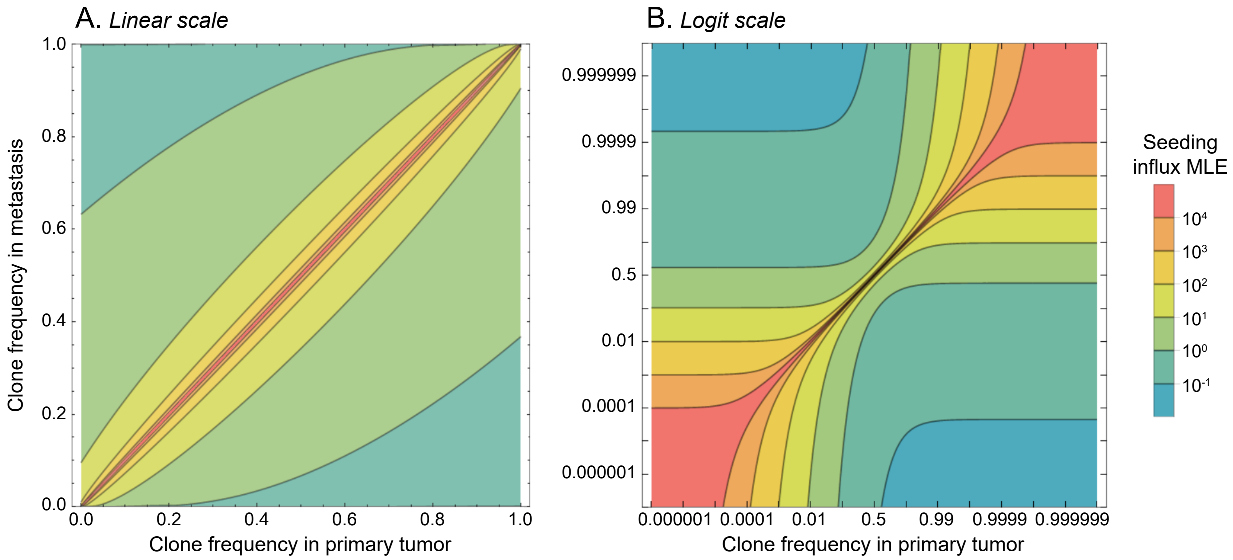

Supplementary Figure 4: MLE inference of seeding influx from a single clone. Given the frequency of a particular clone of interest in a metastasis and its frequency in the primary tumor that seeded it, the maximum likelihood estimate (MLE) for the seeding influx k from the primary tumor to the metastasis is shown using two different axis formats: (A) linear scale, which emphasizes intermediate clone frequencies and (B) logit scale, which emphasizes extreme clone frequencies near 0 or 1.

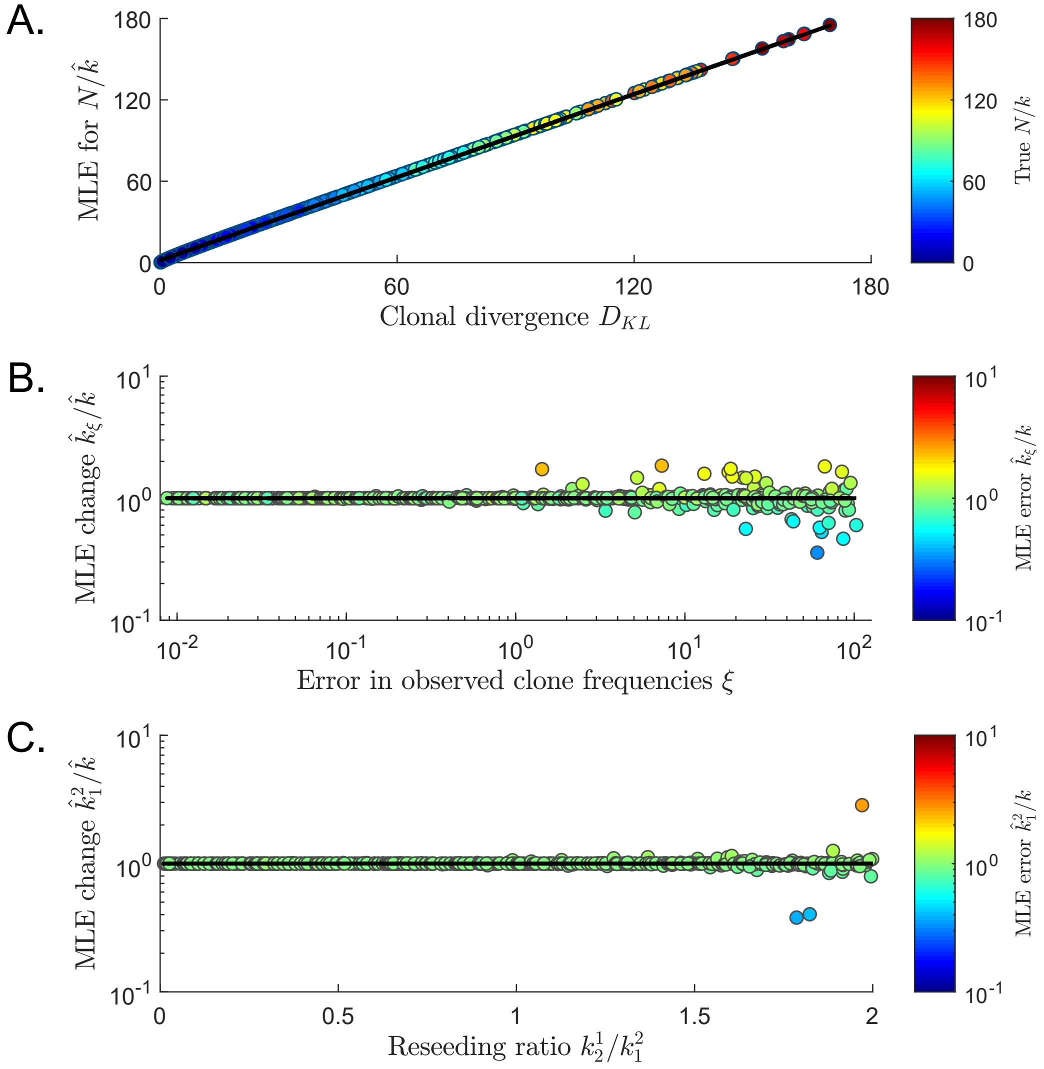

Supplementary Figure 5: Scaling and robustness of MLE seeding influx. Our inference approach computes the MLE seeding influx $\hat{k}$ for a test dataset of 1,000 independently simulated tumor samples with $N = 100$ clones, each with a true seeding influx drawn in the range $k = 10^{-3}$ to $k = 10^3$. (A) Both the average per-clone MLE seeding influx $\hat{k}/N$ and its true value k/N scale inversely with the KL divergence $D_{KL}(\bar{\gamma}\|\gamma)$ between the clonal composition of the two tumors. (B) The MLE seeding influx $\hat{k}_\xi$ was estimated after exposing the test dataset to multiplicative error with standard deviation ξ, and the MLE change $\hat{k}_\xi/\hat{k}$ and MLE error $\hat{k}_\xi/k$ were nearly 1 for all error levels $\xi < 1$. (C) In simulations in which cells could leave the metastasis and reseed the primary tumor with influx k_2^1, where the magnitude of reseeding is measured relative to the forward seeding influx k_1^2 by the reseeding ratio k_2^1/k_1^2, we find that the forward MLE seeding influx $\hat{k}_1^2$ is relatively unchanged compared to its value $\hat{k}$ estimated without reseeding, and the MLE error ratio $\hat{k}_1^2/k$ is generally negligible.

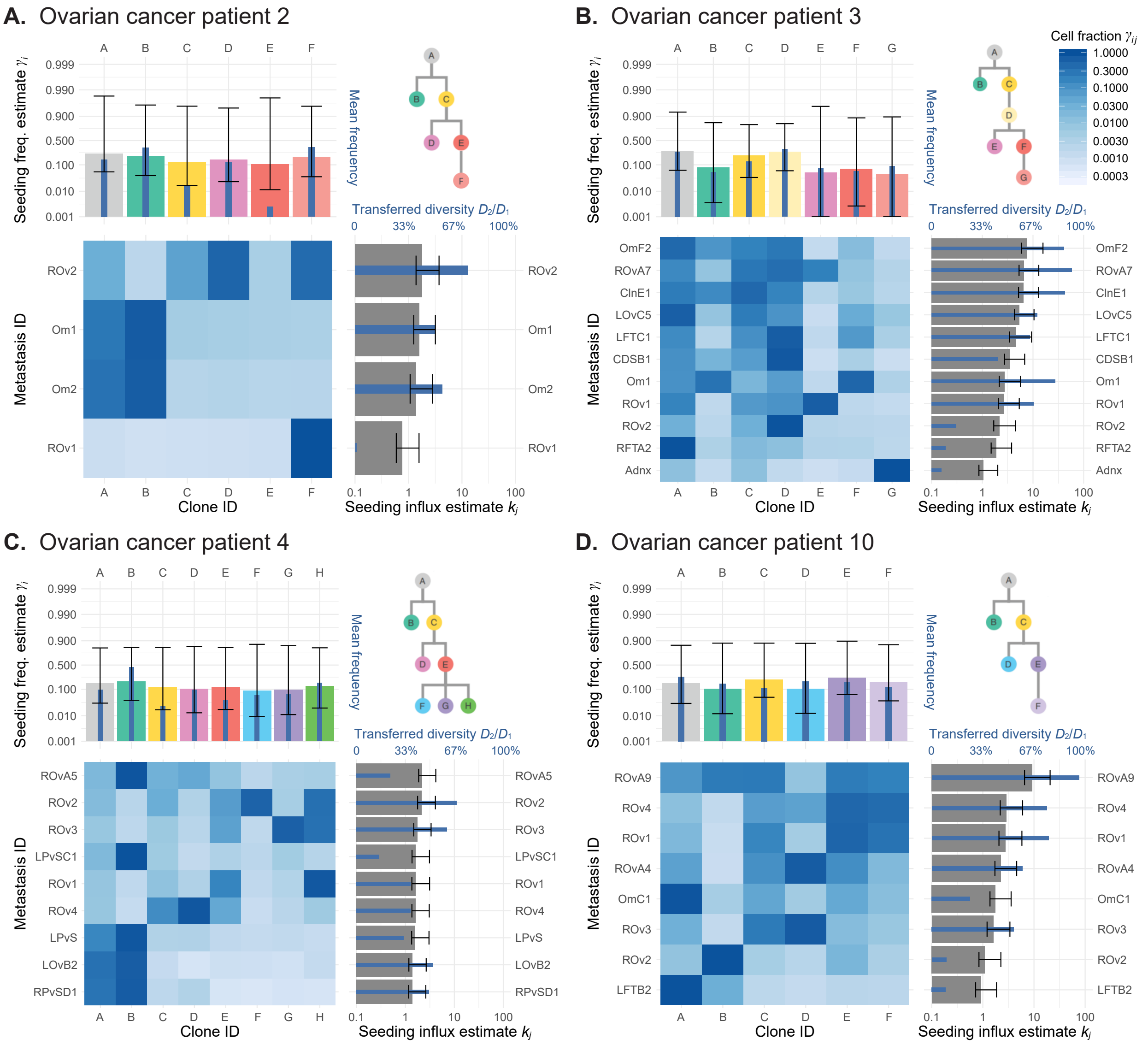

Supplementary Figure 6: Inference of seeding influx in four additional high-grade serous ovarian cancer patients. (A-D) In each panel, the heatmap shows the clonal composition of several sequenced tumors in a patient with high-grade serous ovarian cancer (colorkey top right), as reported by McPherson et al. [1]. As estimated by MLE over the distribution given by Eq. (6) in the main text, the circulating clone frequencies $\bar{\gamma}_i$ of each clone i are depicted as wide colored bars in the top bar chart, and the seeding influxes k_j for each tumor j (the mean number of arriving cells per generation time) as wide gray bars in the right bar chart, with black standard error bars. In addition, the narrow blue bars depict the mean clone frequencies across all metastases (top bar chart) and the fraction of diversity transferred from the circulating cells to each metastasis (right bar chart). The tree in each panel depicts the inferred phylogenetic relationship of the detected clones in the patient.

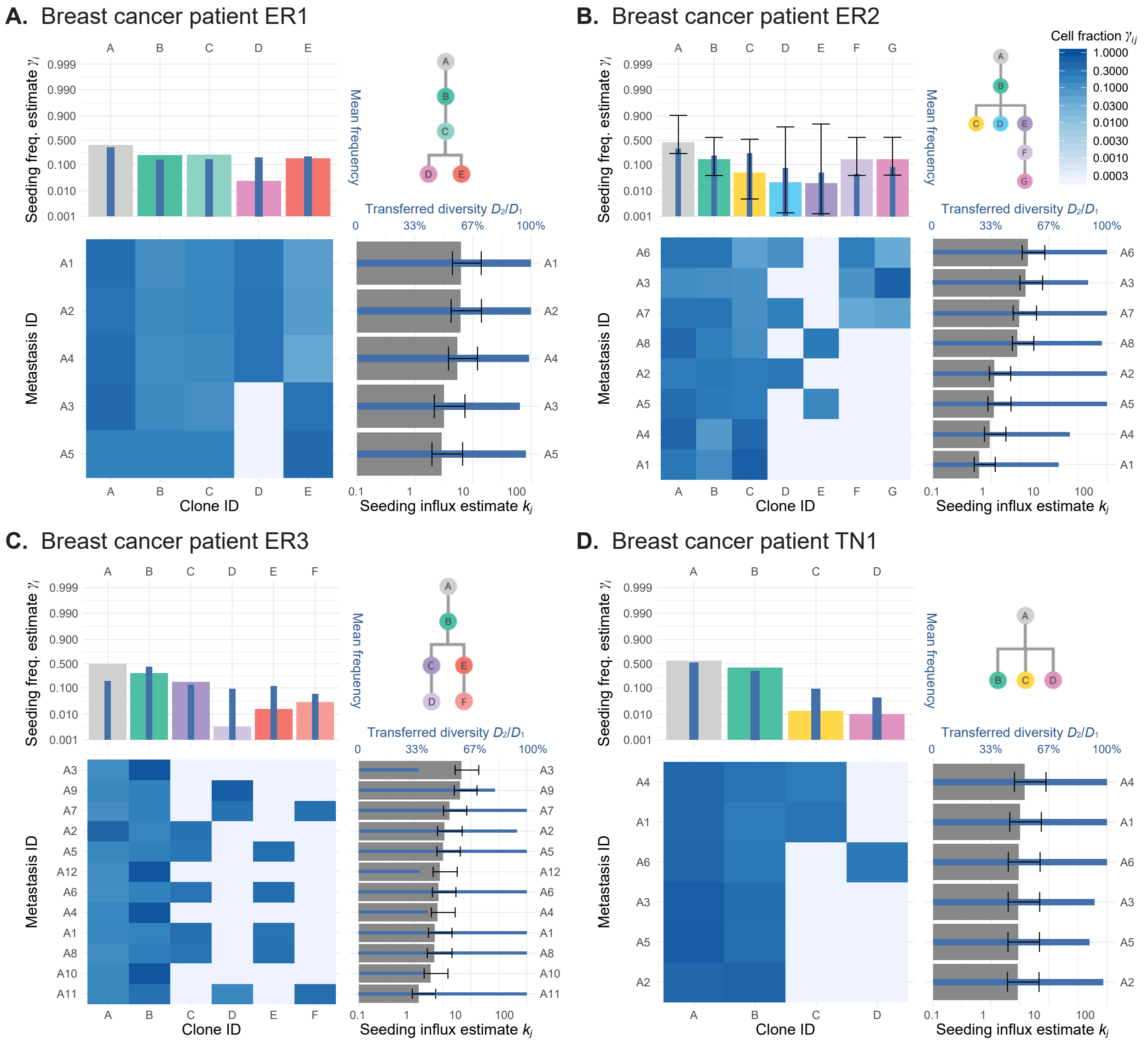

Supplementary Figure 7: Inference of seeding influx in four additional high-grade serous ovarian cancer patients. (A-D) In each panel, the heatmap shows the clonal composition of several sequenced tumors in a patient with breast cancer (colorkey at top right), as reported by Savas et al. [2]. As estimated by MLE over the distribution given by Eq. (6) in the main text, the circulating clone frequencies $\bar{\gamma}_i$ of each clone i are depicted as wide colored bars in the top bar chart, and the seeding influxes k_j for each tumor j (the mean number of arriving cells per generation time) as wide gray bars in the right bar chart, with black standard error bars. In addition, the narrow blue bars depict the mean clone frequencies across all metastases (top bar chart) and the fraction of diversity transferred from the circulating cells to each metastasis (right bar chart). The tree in each panel depicts the inferred phylogenetic relationship of the detected clones in the patient.

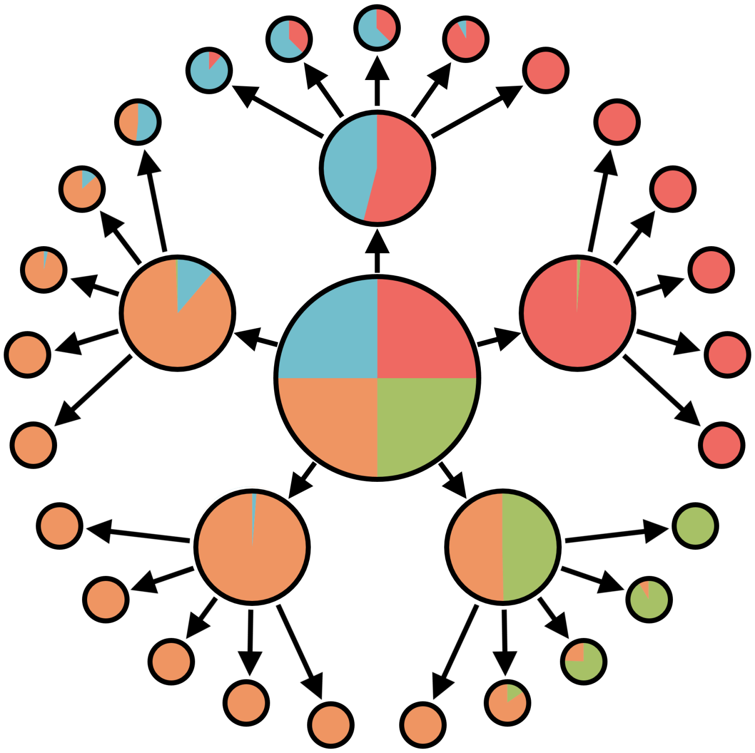

Supplementary Figure 8: Dissemination of clonal diversity via intermediate populations. We simulate our model framework for a primary tumor (large center circle) composed of four neutral clones of equal size at maturity. This primary tumor seeds five secondary populations (inner ring) with a mean influx of $k = 0.33$, sampling from the clonal frequencies of the primary tumor. Once mature, each secondary population seeds five tertiary tumors (outer ring) with the same mean influx $k = 0.33$. For these values, more than 99% of secondary tumors are polyclonal, but only 40% of tertiary tumors are polyclonal.

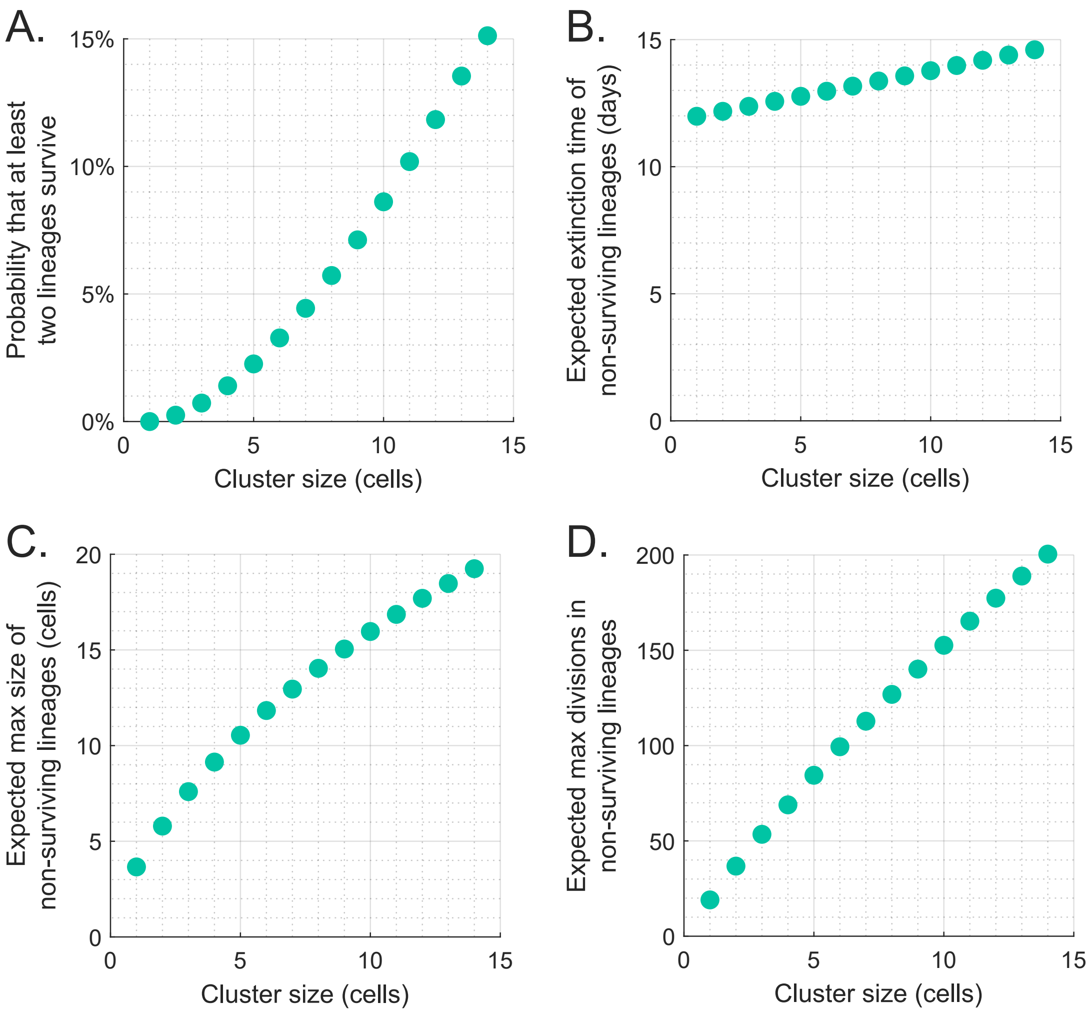

Supplementary Figure 9: Properties of cluster seeding. If a cluster of 1-14 cells were to seed a tumor in our model, typically only 0-1 cells would produce surviving lineages, while the others would produce non-surviving lineages that go extinct quickly. (A) The probability that at least two cell lineages survive in the long run increases with the initial cluster size. For a typical cluster size of 6 cells, this probability is just 3.3%. (B) The expected time until all non-surviving cell lineages go extinct is just 12-15 days, using a growth rate $r = 0.0125$ days^{-1}. (C) A non-surviving lineage might expand in the short run before it goes extinct, but on average only to a maximum of a few dozen cells at most, plotted here as an increasing function of the initially seeded cluster size. (D) Before its extinction, even the non-surviving lineage that divides the most has on average no more than 200 divisions. In contrast, the surviving lineages must divide far more than $Y = 10^8$ times. For all panels, we use $\rho = 5.0\%$ and average over 10^6 independent numerical simulations.

Supplementary Table 1: Seeding influx and seeding frequency estimates by patient ID

Patient ID	Seeding influxes k_j (migrants per generation time) and frequencies $\bar{\gamma}_i$ (%)
Ovarian 1	k_j : 2.12, 2.23, 3.17, 2.02, 1.94, 1.91, 1.58, 1.70, 1.71, 1.76, 2.21, 2.39<br>$\bar{\gamma}_i$: 17.5, 17.0, 9.5, 10.2, 8.4, 9.0, 8.2, 10.8, 9.3
Ovarian 2	k_j : 2.00, 1.74, 0.96, 2.27<br>$\bar{\gamma}_i$: 23.3, 19.7, 12.7, 15.0, 10.6, 18.7
Ovarian 3	k_j : 1.31, 4.34, 8.16, 5.70, 6.72, 3.51, 9.63, 2.38, 3.33, 2.75, 8.30<br>$\bar{\gamma}_i$: 27.4, 8.1, 20.4, 26.9, 5.2, 7.2, 4.7
Ovarian 4	k_j : 1.77, 2.02, 2.06, 2.06, 2.70, 2.23, 2.04, 2.79, 1.74<br>$\bar{\gamma}_i$: 15.9, 18.1, 11.9, 10.5, 12.1, 9.0, 9.7, 12.8
Ovarian 7	k_j : 0.70, 0.79, 1.68, 0.76, 0.79, 1.41, 0.97, 1.17, 1.16, 0.96, 1.99, 1.67, 3.65<br>$\bar{\gamma}_i$: 24.2, 15.1, 27.1, 20.4, 13.0
Ovarian 9	k_j : 0.68, 10.00, 0.65, 0.63, 0.66<br>$\bar{\gamma}_i$: 26.0, 40.0, 34.4
Ovarian 10	k_j : 1.16, 2.22, 3.46, 1.38, 2.02, 3.62, 2.81, 11.62<br>$\bar{\gamma}_i$: 16.0, 10.3, 21.2, 10.4, 24.1, 18.0
Breast ER1	k_j : 7.86, 7.69, 3.97, 6.75, 3.62<br>$\bar{\gamma}_i$: 39.6, 20.3, 21.6, 2.4, 16.0
Breast ER2	k_j : 0.78, 1.43, 4.94, 1.18, 1.40, 5.41, 3.83, 3.57<br>$\bar{\gamma}_i$: 45.8, 14.9, 5.0, 2.2, 2.0, 15.0, 15.2
Breast ER3	k_j : 2.85, 4.22, 8.52, 3.22, 4.01, 3.34, 5.24, 2.77, 8.07, 2.39, 1.46, 3.48<br>$\bar{\gamma}_i$: 49.3, 30.0, 15.9, 0.3, 1.6, 3.0
Breast TN1	k_j : 3.94, 3.59, 3.70, 4.74, 3.67, 3.74<br>$\bar{\gamma}_i$: 56.8, 40.1, 1.3, 1.0
Colorectal A01	k_j : 1.40<br>$\bar{\gamma}_i$: 30.6, 13.8, 38.5, 13.7, 3.3
Colorectal A02	k_j : 7.15<br>$\bar{\gamma}_i$: 39.4, 22.7, 16.7, 21.2
Colorectal A03	k_j : 114.5<br>$\bar{\gamma}_i$: 67.3, 22.2, 10.5
Colorectal A04	k_j : 5.85<br>$\bar{\gamma}_i$: 30.8, 10.3, 24.2, 23.7, 11.0

Estimates for all seeding influxes k_j, defined as the mean number of cells that migrate to metastasis j per cell generation, and all seeding frequencies $\bar{\gamma}_i$, defined as the mean number of migrating cells descended from clone i, for each patient included in our analysis. Estimates were obtained by maximum likelihood estimation using the consecutive seeding model.

Supplementary Analysis

We consider a growing metastasis composed of $y(t)$ total cells at time t [3, 4, 5, 6, 7]. Each cell in the metastasis derives from one of N clones originating in the primary tumor, and we let $y_i(t)$ denote the number of cells derived from clone $i = 1, \dots, N$ in the metastasis at time t (Fig. S1). Cells from each clone i arrive at the metastasis with a constant seeding rate λ_i, which can be considered the product of three factors: the frequency of the clone in the primary tumor, the total size of the primary tumor, and the average per-cell likelihood of metastasis from the primary tumor. This latter factor may be affected, for example, by the clonal genotype or the spatial arrangement of clones in the primary tumor. Consequently, we avoid strict assumptions about the magnitude of λ_i, allowing for wide-ranging seeding rates. The total seeding rate across all clones is denoted by $\lambda = \sum_{i=1}^{N} \lambda_i$.

Once at the metastasis site, each cell of clone i replicates according to an exponential birth-death process with division rate b_i and death rate d_i [8, 9]. Each clone i has a positive net growth rate $r_i = b_i - d_i$, and the average number of clone i migrants per generation is given by the seeding influx $k_i = \lambda_i / b_i$. The probability that a cell gives rise to a lineage that survives is $\rho_i = 1 - d_i/b_i$, and the average number of lifetime divisions of each cell is $(1-\rho_i)^{-1} = b_i/d_i$. Overall, each clone varies in size with three degrees of freedom, either expressed as the model rates (b_i, d_i, λ_i), or more conveniently, the model parameters (r_i, ρ_i, k_i). If time is measured in units of average generation time such that $b_i = 1$, then note that $\rho_i = r_i$ and $k_i = \lambda_i$. Several of our derivations consider the special case of neutral passenger diversity, such that $b_i = b$ and $d_i = d$ for all clones i, but the rates λ_i can freely vary between clones [10, 11, 12, 13].

We initialize the metastasis to have zero size at time $t = 0$, such that $y_i(0) = 0$ for all clones i. The detection time T can then be defined as the first time that the total metastasis size $y(t)$ reaches some fixed detection size Y, in terms of the average number of all migrants per generation, $k = \sum_{i=1}^{N} k_i = \lambda/b$.

Proposition 1. The mean size $\bar{y}_i(t)$ of each clone at time t is $\bar{y}_i(t) = \frac{\lambda_i}{r_i}(e^{r_i t} - 1)$.

Proof: If there are $y_i(t)$ cells derived from clone i at time t, then the number of cells $y_i(t+\tau)$ derived from clone i at time $t + \tau$ is given by

$$y_i(t+\tau) = y_i(t) + B_i(t, t+\tau) - D_i(t, t+\tau) + L_i(t, t+\tau), \tag{1}$$

where the numbers of births B_i and deaths D_i between time t and time $t + \tau$, in the limit as $\tau \to 0^+$, follow a binomial distribution in which each of the $y_i(t)$ cells has a probability $b_i\tau$ to divide and $d_i\tau$ to die, each being sufficiently small to permit a Poisson approximation:

$$B_i(t, t+\tau) \sim \text{Bin}(y_i(t), b_i\tau) \to \text{Pois}(y_i(t) b_i \tau), \tag{2}$$

$$D_i(t, t+\tau) \sim \text{Bin}(y_i(t), d_i\tau) \to \text{Pois}(y_i(t) d_i \tau), \tag{3}$$

and the number of migrants L_i between time t and time $t + \tau$ follows a binomial distribution in which each of the x_i cells of clone i in the primary tumor has a probability $\ell_i\tau$ to migrate:

$$L_i(t, t+\tau) \sim \text{Bin}(x_i, \ell_i\tau) \to \text{Pois}(x_i \ell_i \tau). \tag{4}$$

Taking the expectation of both sides of equation 1 gives

$$\bar{y}_i(t+\tau) = \bar{y}_i(t) + \bar{y}_i(t)b_i\tau - \bar{y}_i(t)d_i\tau + \lambda_i\tau, \tag{5}$$

where the seeding rate is defined as $\lambda_i = x_i\ell_i$. Rearranging, we obtain

$$\frac{\bar{y}_i(t+\tau) - \bar{y}_i(t)}{\tau} = r_i\bar{y}_i(t) + \lambda_i \tag{6}$$

since $r_i = b_i - d_i$, and taking the limit as $\tau \to 0^+$ gives the rate of clone size expansion,

$$\dot{\bar{y}}_i(t) = r_i\bar{y}_i(t) + \lambda_i. \tag{7}$$

where the overset dot denotes the time derivative. The solution of this differential equation for the clone size $\bar{y}_i(t)$ is given by

$$\bar{y}_i(t) = \frac{\lambda_i}{r_i}(e^{r_it} - 1) = \frac{k_i}{\rho_i}(e^{r_it} - 1), \tag{8}$$

where the initial condition of no cells at the metastatic site, $\bar{y}_i(0) = 0$, has been applied.

Proposition 2. The size $y_i(t)$ of the ith clone follows the negative binomial distribution

$$y_i(t) \sim \text{NBin}\left(k_i = \frac{\lambda_i}{b_i},\ q_i(t) = \frac{k_i}{\bar{y}_i(t) + k_i} = \frac{\rho_i}{e^{r_it} - (1-\rho_i)}\right). \tag{9}$$

Proof: The master equation that governs the probability P_i that the ith clone size is equal to y_i at time t can be written as

$$\dot{P}_i(y_i|t) = \big[\lambda_i + b_i(y_i-1)\big]P_i(y_i-1|t) - \big[\lambda_i + (b_i+d_i)y_i\big]P_i(y_i|t) + \big[d_i(y_i+1)\big]P_i(y_i+1|t), \tag{10}$$

where we enforce the normalization condition $\sum_{y_i=0}^{\infty} P_i(y_i|t) = 1$ and the boundary condition $P_i(-1|t) = 0$, because clone sizes y_i must be non-negative integers. This master equation can be rewritten in terms of the backward step operator S, defined such that $S^k[f(y_i)] = f(y_i - k)$:

$$\dot{P}_i(y_i|t) = (S-1)\big[(\lambda_i + b_iy_i)P_i(y_i|t)\big] + (S^{-1}-1)\big[d_iy_iP_i(y_i|t)\big]. \tag{11}$$

It is useful to again rewrite equation 11 in terms of the probability generating function of the clone size, defined mathematically as $G_i(s|t) = \mathbb{E}[s^{y_i}|t] = \sum_{y_i=0}^{\infty} s^y P_i(y_i|t)$, which gives

$$\dot{G}_i(s|t) = (s-1)\big[\lambda_iG_i(s|t) + b_isG_i'(s|t)\big] + (s^{-1}-1)\big[d_isG_i'(s|t)\big]. \tag{12}$$

where a prime denotes differentiation with respect to s. This first-order linear partial differential equation can be solved by the method of characteristics, yielding the solution

$$G_i(s|t) = \left[\frac{\rho_i}{s + (1-s)e^{r_it} - (1-\rho_i)}\right]^{\frac{\lambda_i}{b_i}} = \left[\frac{q_i(t)}{1 - s(1-q_i(t))}\right]^{k_i}, \tag{13}$$

where $\rho_i = 1 - d_i/b_i$, and k_i and q_i are defined as in equation 9. We recognize that $G_i(s|t)$ is the generating function of a negative binomial random variable with parameters k_i and q_i, with the associated probability mass function for the clone size $y_i(t)$ given by

$$P_i(y_i|t) = \binom{y_i + k_i - 1}{y_i} q_i(t)^{k_i} \big[1 - q_i(t)\big]^{y_i} \tag{14}$$

over the support of non-negative integers y_i, which hence solves the master equation 11. The first factor in this expression is a binomial coefficient and can be calculated even if the mean number of migrants per generation k_i is not an integer by extending the factorial in the usual manner by using the gamma function, so that

$$P_i(y_i|t) = \frac{\Gamma(y_i + k_i)}{y_i!\,\Gamma(k_i)}\, q_i(t)^{k_i} \big[1 - q_i(t)\big]^{y_i}. \tag{15}$$

Corollary 2.1. The coefficient of variation in clone size converges to $\sqrt{1/k_i}$ over time.

Proof: The variance in clone size y_i at time t can be derived from its probability generating function $G_i(s|t)$ by computing the quantity

$$\mathrm{Var}[y_i|t] = G_i''(1|t) + G_i'(1|t) - G_i'(1|t)^2 \tag{16}$$

Computing this value using the probability generating function found in equation 12 gives

$$\mathrm{Var}[y_i|t] = \frac{\bar{y}_i(t)}{q_i(t)} = \frac{k_i}{\rho_i^2}\big(e^{r_i t} - 1\big)\big(e^{r_i t} - 1 + \rho_i\big) \tag{17}$$

Using the mean from Proposition 1, the coefficient of variation in clone size is then

$$\frac{\sqrt{\mathrm{Var}[y_i|t]}}{\bar{y}_i(t)} = \sqrt{\frac{1}{k_i} \times \frac{e^{r_i t} - 1 + \rho_i}{e^{r_i t} - 1}} \tag{18}$$

Over time, for $e^{r_i t} \gg 1$, this coefficient of variation rapidly converges to $\sqrt{1/k_i}$.

Corollary 2.2. The size of a metastasis y_i at time t can be equivalently generated via a repeated Bernoulli process. Beginning with an empty site for the metastasis,

1. Create a pool of k_i circulating cells and $\bar{y}_i$ potential daughter cells.

2. Choose a cell uniformly at random from this pool and add a copy of it to the metastasis. If, with probability $q_i = \frac{k_i}{k_i + \bar{y}_i}$, a circulating cell is chosen, then remove a cell at random from the metastasis.

3. Repeat step 2 until k_i circulating cells have been chosen from the pool. The number of cells in the metastasis will then be distributed as desired, $y_i \sim \mathrm{NBin}(k_i, q_i)$.

Proof: Arbitrarily, we will refer to the event that a circulating cell is chosen as a "failure" and the event that a daughter cell is chosen as a "success." The number of cells y_i in the metastasis following the process described here is then the number of successes before k_i failures occur, each with failure probability q_i. This is one standard definition of the negative binomial distribution.

To show that this is equivalent, we can compute the probability that there are y_i successes and $k_i - 1$ failures before the k_ith failure. There are $\binom{y_i+k_i-1}{y_i}$ ways to choose the position of the $k_i - 1$ failures in the string of the first $y_i + k_i - 1$ outcomes, and the probability of each of these strings is the product of the probability of each outcome: $q_i^{k_i-1}(1-q_i)^{y_i}$. The $y_i + k_i$ trial will then fail with probability q_i. Therefore the probability that the first $y_i + k_i - 1$ outcomes will have $k_i - 1$ failures and the k_ith outcome will be a failure is

$$P_i(y_i|t) = \binom{y_i + k_i - 1}{y_i} q_i^{k_i}(1 - q_i)^{y_i} \tag{19}$$

This precisely matches equation 14, and it is the probability mass function of a variable with a negative binomial distribution, $y_i \sim \text{NBin}(k_i, q_i)$.

Corollary 2.3. The number of surviving lineages $L_i(t)$ at time t, each originated by a unique seeding event, follows a Poisson distribution with mean $-k_i \ln q_i(t)$.

Proof: Because the seeding rate is assumed to be constant in time, the number of seeding events will be Poisson-distributed with mean $\lambda_i t$. The probability that each lineage will survive until a time τ after it arrives is given by

$$G_i(0|\tau) = \frac{\rho_i}{1 - (1 - \rho_i)e^{-r_i\tau}} \tag{20}$$

The number of surviving lineages at time t is then Poisson-distributed with mean

$$\int_0^t \lambda_i \, G_i(0|\tau)\, d\tau = \frac{\lambda_i\rho_i}{r_i} \ln\left(1 + \rho_i^{-1}(e^{r_i t} - 1)\right) = -k_i \ln q_i(t) \tag{21}$$

which is approximately $\lambda_i t$ at small times $t \ll 1/r_i$ and approximately $\lambda_i\rho_i t - k_i \ln \rho_i$ at large times $t \gg 1/r_i$.

Corollary 2.4. The size of each lineage at time t follows a log-series distribution $x_{ij}(t) \sim \text{LogS}(1 - q_i(t))$, with probability mass function

$$P_{ij}(x_{ij}|t) = -\frac{[1 - q_i(t)]^{x_{ij}}}{x_{ij} \ln q_i(t)} \tag{22}$$

Proof: Define the probability generating functions of the number of surviving lineages and the size of each surviving lineage as $G_i^L(s|t) = \mathbb{E}[s^{L_i}|t]$ and $G_i^x(s|t) = \mathbb{E}[s^{x_{ij}}|t]$. The generating function of a $\text{Pois}(\mu)$ random variable is $e^{-\mu(1-s)}$, so the generating function of

$L \sim \text{Pois}(-k_i \ln q_i(t))$ is $G_i^L(s|t) = q_i^{k_i(1-s)}$. Because these variables are related as

$$y_i = \sum_{j=1}^{L_i} x_{ij} \tag{23}$$

we have that $G_i(s|t) = \mathbb{E}[s^{y_i}|t] = G_i^L(G_i^x(s|t)|t)$. Using the generating function of $y_i(t)$ from equation 13, this is equivalent to

$$\left(\frac{q_i(t)}{1 - s(1 - q_i(t))}\right)^{k_i} = q_i(t)^{k_i\left(1 - G_i^x(s|t)\right)} \tag{24}$$

This directly implies that

$$G_i^x(s|t) = \log_{q_i(t)} \Big[1 - s\Big(1 - q_i(t)\Big)\Big] \tag{25}$$

which is precisely the probability generating function of the $\text{LogS}(1 - q_i(t))$ distribution.

Lemma 2.6. For sufficiently long times $rt \gg 1$, the deviation from expected size $\hat{y}_i(t)$ converges to a gamma distribution,

$$\hat{y}_i(t) = \frac{y_i(t)}{\bar{y}_i(t)} \sim \text{Gamma}(k_i, k_i) \tag{26}$$

Proof: To describe the shape of the clone size distribution in the limit of long times, we consider the distribution of $\hat{y}_i = y_i/\bar{y}_i$ in the limit $t \to \infty$. The probability density function $f_i(\hat{y}_i)$ of $\hat{y}_i$ can be calculated as follows:

$$f_i(\hat{y}_i) = \lim_{t\to\infty} \Big[\bar{y}_i P_i(\bar{y}_i\hat{y}_i|t)\Big] \tag{27}$$

$$= \frac{1}{\hat{y}_i\,\Gamma(k_i)} \lim_{t\to\infty} \Big[(\bar{y}_i\hat{y}_i)^{(k_i)}\,(1 - q_i)^{\bar{y}_i\hat{y}_i}\,(1 - p_i)^{k_i}\Big] \tag{28}$$

$$= \frac{1}{\hat{y}_i\,\Gamma(k_i)} \exp\Big(\lim_{t\to\infty} \big[\ln(\bar{y}_i\hat{y}_i)^{(k_i)} + k_i \ln q_i + \bar{y}_i\hat{y}_i \ln(1 - q_i)\big]\Big) \tag{29}$$

$$= \frac{1}{\hat{y}_i\,\Gamma(k_i)} \exp\Big(k_i \ln \tfrac{\lambda_i \hat{y}_i}{r_i} + k_i \ln \tfrac{r_i}{b_i} - k_i\hat{y}_i\Big) \tag{30}$$

$$= \frac{k_i^{k_i}}{\Gamma(k_i)}\,\hat{y}_i^{k_i - 1} e^{-k_i\hat{y}_i} \tag{31}$$

This is the probability density function of a $\text{Gamma}(k_i, k_i)$ random variable.

Proposition 3. If the detection size Y is sufficiently large, then the detection time T follows a log-gamma distribution with mean $\bar{T}$ and mode $\hat{T}$ equal to

$$\bar{T} = r^{-1}\big[\ln(\rho Y) - \psi(k)\big] \tag{32}$$

$$\hat{T} = r^{-1}\big[\ln(\rho Y) - \ln(k)\big] \tag{33}$$

Proof: If the detection size Y, and hence also the detection time T, is sufficiently large that $rT \gg 1$, then Lemma 2.6 gives that $\hat{y}(T) = y(T)/\bar{y}(T)$ approximately follows a Gamma(k, k) distribution. We know that $y(T) = Y$ and we know $\bar{y}(T)$ from Proposition 1, so

$$\hat{y}(T) = \frac{Y}{\frac{\lambda}{r}(e^{rT} - 1)} \sim \text{Gamma}(k, k) \tag{34}$$

Since $rT \gg 1$, then this is approximately $\frac{Yr}{\lambda} e^{-rT} \sim \text{Gamma}(k, k)$. We define $\omega = e^{-rT}$, and applying the scaling property gives that $\omega = e^{-rT} \sim \text{Gamma}(k, \rho Y)$. Using the change of variables $T = -r^{-1} \ln \omega$ gives the probability density function of the detection time T,

$$f_T(t) = \frac{r(\rho Y)^k}{\Gamma(k)} \exp\big\{ - krt - \rho Y e^{-rt} \big\} \tag{35}$$

corresponding to a log-gamma distribution. To compute the mean detection time, we note that $\bar{T} = -r^{-1}\mathbb{E}[\ln \omega]$, and since ω follows the gamma distribution as noted above, we can apply the known expression for the mean of the logarithm of a gamma random variable:

$$\bar{T} = -r^{-1}\mathbb{E}[\ln \omega] = r^{-1}\big[\ln(\rho Y) - \psi(k)\big] \tag{36}$$

where $\psi(x) = \frac{d}{dx} \ln \Gamma(x)$ is the digamma function. In addition, the mode detection time $\hat{T}$ can be directly computed by solving $\frac{d}{dt} f_T(t) = 0$ to give

$$\hat{T} = r^{-1} \ln \frac{rY}{\lambda} = r^{-1}\big[\ln(\rho Y) - \ln(k)\big]. \tag{37}$$

Corollary 3.1. *The difference between the mean and mode detection times, $\bar{T} - \hat{T}$, scales as $(rk)^{-1}$ for small k but scales as $(2rk)^{-1}$ for large k.*

Proof: The difference between the mean and mode detection times is

$$\bar{T} - \hat{T} = r^{-1}\big[\ln(k) - \psi(k)\big] \tag{38}$$

Expanding this expression about $k = 0$ gives

$$\bar{T} - \hat{T} = \frac{1}{rk} + \frac{c_\gamma + \ln k}{r} - \frac{\pi^2 k}{6r} + O(k^2) \tag{39}$$

where $c_\gamma \approx 0.577$ is the Euler-Mascheroni constant and $\pi \approx 3.14$ is the Archimedes constant. For very small $k \ll 1$, the $(rk)^{-1}$ term dominates, and so approximately $\bar{T} - \hat{T} = (rk)^{-1}$. Hence, in the limit $k \to 0$, we have that $\bar{T} - \hat{T} \to \infty$.
If we instead expand the expression for the difference about $k = \infty$, we obtain

$$\bar{T} - \hat{T} = \frac{1}{2rk} + \frac{1}{12rk^2} + O(k^{-3}) \tag{40}$$

and for very large $k \gg 1$, the $(2rk)^{-1}$ term dominates, so approximately $\bar{T} - \hat{T} = (2rk)^{-1}$. Hence, in the limit $k \to \infty$, we have that $\bar{T} - \hat{T} \to 0$.

Corollary 3.2. The mean detection time overestimates the time at which the average tumor size reaches the detection threshold, $\bar{y}(\bar{T}) > Y$, while the mode accurately reflects this timing, $\bar{y}(\hat{T}) = Y$.

Proof: The average tumor size at the mode detection time can be computed as

$$\bar{y}(\hat{T}) \approx \frac{k}{\rho} \cdot e^{r\hat{T}} = \frac{k}{\rho} \cdot \frac{\rho Y}{k} = Y \tag{41}$$

where the approximation assumes that $r\hat{T} \gg 0$. In contrast, for the mean detection time,

$$\bar{y}(\bar{T}) \approx \frac{k}{\rho} \cdot e^{r\bar{T}} = Y e^{\ln(k) - \psi(k)} > Y \tag{42}$$

where we have used the fact that $\ln(k) > \psi(k)$ from Corollary 3.1.

Corollary 3.3. The expected number of cells that migrated to the metastasis prior to detection and gave rise to a surviving lineage is given by

$$\bar{X} = \rho\lambda\bar{T} = k\Big[\ln(\rho Y) - \psi(k)\Big]. \tag{43}$$

Proof: The expected number of cells that migrate to the metastasis over a time period τ is $\lambda\tau$, and the expected fraction of those cells that give rise to a surviving lineage is ρ. Hence the expected number of cells that migrate during the time period T before detection and give rise to a surviving lineage is

$$\mathbb{E}[X] = \mathbb{E}[\lambda\rho T] = \lambda\rho\bar{T} = k\Big[\ln(\rho Y) - \psi(k)\Big] \tag{44}$$

which is precisely the result to be obtained.

Proposition 4. In a tumor of size Y, the vector of neutral clone sizes $(Y_1, \dots, Y_N)$, which obey $Y = \sum_{i=1}^{N} Y_i$, follows a Dirichlet-multinomial distribution, with mass function

$$P(Y_1, \dots, Y_N) = \frac{\prod_{i=1}^{N} \binom{Y_i + k_i - 1}{Y_i}}{\binom{Y+k-1}{Y}} \tag{45}$$

Proof: Let ΣY_i denote the sum of the first i clone sizes, $\sum_{j=1}^{i} Y_j$, and let Σk_i denote the sum of the first i seeding ratios, $\sum_{j=1}^{i} k_j$. The distribution of ΣY_i is proportional to

$$P(\Sigma Y_i) \propto \binom{\Sigma Y_i + \Sigma k_i - 1}{\Sigma Y_i} \tag{46}$$

since the first n clones can be treated as a single superclone with total size ΣY_i and seeding ratio Σk_i. If the nth clone size is known, then this clone can be excluded from the superclone, and so the superclone has only total size ΣY_{i-1} and seeding ratio Σk_{i-1}. Mathematically,

$$P(\Sigma Y_i | Y_i) \propto \binom{\Sigma Y_i - Y_i + \Sigma k_i - k_i - 1}{\Sigma Y_i - Y_i} = \binom{\Sigma Y_{i-1} + \Sigma k_{i-1} - 1}{\Sigma Y_{i-1}} \tag{47}$$

Conversely, the marginal distribution of the ith clone size Y_i given $Y = \Sigma Y_i$ can be found using Bayes' law, where the proportionality factors can be eliminated to give

$$P(Y_i|\Sigma Y_i) = \frac{P(\Sigma Y_i|Y_i)}{P(\Sigma Y_i)}\, P(Y_i) = \frac{\binom{\Sigma Y_{i-1}+\Sigma k_{i-1}-1}{\Sigma Y_{i-1}}}{\binom{\Sigma Y_i+\Sigma k_i-1}{\Sigma Y_i}} \binom{Y_i + k_i - 1}{Y_i} \tag{48}$$

which is the mass function of a Beta-binomial distribution. Now we can compute the full joint mass function of Y_2 to Y_N (which also specifies that $Y_1 = Y - Y_N - \cdots - Y_2$),

$$P(Y_1, \ldots, Y_N|\Sigma Y_N) = \prod_{i=2}^{N} P(Y_i|\Sigma Y_i) = \frac{\binom{\Sigma Y_1+\Sigma k_1-1}{\Sigma Y_1}}{\binom{\Sigma Y_N+\Sigma k_N-1}{\Sigma Y_N}} \prod_{i=2}^{N} \binom{Y_N + k_N - 1}{Y_N} \tag{49}$$

since many of the binomial coefficients balance with the neighboring factors in the product. Then, noting that $\Sigma Y_1 = Y_1$, $\Sigma k_1 = k_1$, $\Sigma Y_N = Y$, and $\Sigma k_N = k$, we obtain the result

$$P(Y_1, \ldots, Y_N) = \frac{\prod_{i=1}^{N} \binom{Y_i+k_i-1}{Y_i}}{\binom{Y+k-1}{Y}} \tag{50}$$

where we need not condition on ΣY_N because it must be equal to the known tumor size Y.

Corollary 4.1. The mean number of clones n in the metastasis is given by

$$\bar{n} = N - \frac{\sum_{i=1}^{N} \binom{Y+k-k_i-1}{Y}}{\binom{Y+k-1}{Y}} \tag{51}$$

Proof: Consider only the joint distribution of the ith clone size Y_i and the total number of other cells, which has a superclone size $Y - Y_i$ with seeding ratio $k - k_i$. Using the result from Proposition 4, we know that the joint probability mass function for these two sizes is

$$P(Y_i) = P(Y_i, Y - Y_i) = \frac{\binom{Y_i+k_i-1}{Y_i}\binom{Y-Y_i+k-k_i-1}{Y-Y_i}}{\binom{Y+k-1}{Y}} \tag{52}$$

To find the probability that the ith clone is absent, we evaluate this at $Y_i = 0$ to obtain

$$P(Y_i = 0) = \frac{\binom{Y+k-k_i-1}{Y}}{\binom{Y+k-1}{Y}} \tag{53}$$

The mean number of clones n in the tumor is then given by

$$\bar{n} = \sum_{i=1}^{N} P(Y_i > 0) = \sum_{i=1}^{N} \left[1 - \frac{\binom{Y+k-k_i-1}{Y}}{\binom{Y+k-1}{Y}}\right] \tag{54}$$

which gives precisely the result to be proved.

Corollary 4.2. The probability that a metastasis is polyclonal is

$$P(n > 1) = 1 - \frac{\sum_{i=1}^{N} \binom{Y+k_i-1}{Y}}{\binom{Y+k-1}{Y}} \tag{55}$$

Proof: To find the probability that the ith clone size Y_i is equal to the total tumor size Y, we simply evaluate equation 52 at $Y_i = Y$ to obtain

$$P(Y_i = Y) = \frac{\binom{Y+k_i-1}{Y}}{\binom{Y+k-1}{Y}} \tag{56}$$

The probability that the tumor is monoclonal for any of the clones is then given by the sum $P(n = 1) = \sum_{i=1}^{N} P(Y_i = Y)$, and so the probability of polyclonality is the complement of this sum, which gives precisely the result to be proved.

Corollary 4.3. The probability that exactly n of the N clones from the primary tumor are present at nonzero frequency in a metastasis of size Y is

$$P(n) = B_N^{-1} \sum_{m=1}^{n} \binom{N-m}{N-n} (-1)^{n-m} B_m, \quad \text{where } B_n = \sum_{I_n \in \binom{I}{n}} \binom{Y + \sum_{i \in I_n} k_i - 1}{Y} \tag{57}$$

and $\binom{I}{n}$ is the set of all unordered combinations of length n from the set $I = \{1, 2, \ldots, N\}$.

Proof: The probability that the subset of clones S present in the metastasis is included in any subset of clones $I_n \subseteq I = \{1, 2, \ldots, N\}$ is

$$P(S \subseteq I_n) = \frac{\binom{Y + \sum_{i \in I_n} k_i - 1}{Y}}{\binom{Y+k-1}{Y}}$$

and summing over all sets $I_n \subseteq I$ with n elements, that is over the elements I_n of $\binom{I}{n}$, gives B_n/B_N. We then apply the inclusion-exclusion principle to obtain the distribution of n,

$$P(n) = \sum_{j=1}^{n} \binom{N-n+j-1}{N-n} (-1)^{j-1} \frac{B_{n-j+1}}{B_N} \tag{58}$$

and setting the index of summation to $m = n - j + 1$ gives the desired result.

Corollary 4.4. In the case where all N clones are seeded at an equal and low rate $k_i = k/N \ll 1$ and the tumor size is large, $Y \gg 1$, the polyclonal probability is approximately

$$P(n > 1) \approx 1 - \kappa! \, Y^{-\kappa} \tag{59}$$

where $\kappa = k\left(1 - \frac{1}{N}\right)$ is a clone-adjusted seeding influx.

Proof: Given $k_i = k/N$ for all clones i, the expression for the polyclonality probability from Corollary 4.2 can be written as

$$P(n > 1) = 1 - N\frac{\binom{Y+k/N-1}{Y}}{\binom{Y+k-1}{Y}} \tag{60}$$

For a large tumor size Y, we apply the binomial approximation $\binom{Y+z}{Y} \approx Y^z/\Gamma(z)$ to obtain

$$P(n > 1) \approx 1 - N\,\frac{Y^{k/N}}{Y^k}\frac{\Gamma(k)}{\Gamma(\frac{k}{N})} \tag{61}$$

Given $k/N \ll 1$, we can approximate $\Gamma(k/N) \approx N\Gamma(k)/\Gamma(k - \frac{k}{N} + 1)$. Then we have

$$P(n > 1) \approx 1 - \Gamma(k - \tfrac{k}{N} + 1)Y^{-k\left(1-\frac{1}{N}\right)} = 1 - \Gamma(\kappa + 1)Y^{-\kappa} \tag{62}$$

and because $\Gamma(\kappa + 1) = \kappa!$, this is precisely the result to be proved.

Proposition 5. The vector of clone frequencies $(\gamma_1, \ldots, \gamma_N)$ follows a Dirichlet distribution, with joint probability density function

$$P(\gamma_1, \ldots, \gamma_N) = \Gamma(k)\prod_{i=1}^{N}\frac{\gamma_i^{k_i-1}}{\Gamma(k_i)} \tag{63}$$

Proof: Given the total tumor size Y, we can rewrite the result of claim 4 in terms of the clone frequencies $\gamma_i = Y_i/Y$ by using the change of variables $\vec{Y} = \vec{\gamma}Y$,

$$P(\gamma_1, \ldots, \gamma_N) = \frac{Y\prod_{i=1}^{N}\binom{\gamma_i Y+k_i-1}{\gamma_i Y}}{\binom{Y+k-1}{Y}} \tag{64}$$

Using Stirling's approximation $\ln\Gamma(x) \approx x(\ln x - 1)$ for large x, we can approximate the logarithm of the binomial coefficient for large Y with the expression

$$\ln\binom{Y+k-1}{Y} \approx (Y + k)\big[\ln(Y + k) - 1\big] - (Y + 1)\big[\ln(Y + 1) - 1\big] - \ln\Gamma(k) \tag{65}$$

With this approximation, the logarithm of the joint density function is approximately

$$\ln P(\vec{\gamma}) = \ln Y - \ln\binom{Y+k-1}{Y} + \sum_{i=1}^{N}\ln\binom{\gamma_i Y+k_i-1}{\gamma_i Y} \tag{66}$$

$$\approx \ln Y - (Y + k)\ln(Y + k) + (Y + 1)\ln(Y + 1) - 1 + \ln\Gamma(k) \tag{67}$$

$$+ \sum_{i=1}^{N}\Big[(\gamma_i Y + k_i)\ln(\gamma_i Y + k_i) - (\gamma_i Y + 1)\ln(\gamma_i Y + 1) + 1 - \ln\Gamma(k_i)\Big] \tag{68}$$

$$\approx \ln\Gamma(k) + \sum_{i=1}^{N}\Big[(k_i - 1)\ln\gamma_i - \ln\Gamma(k_i)\Big] \tag{69}$$

provided that $Y \gg 1$. Exponentiating this expression directly gives the result to be proved.

Corollary 5.1. The mean, variance, and covariances in clone frequencies γ_i are given by

$$\bar{\gamma}_i = \frac{k_i}{k}, \qquad \mathrm{Var}[\gamma_i] = \frac{\bar{\gamma}_i(1-\bar{\gamma}_i)}{1+k}, \qquad \mathrm{Cov}[\gamma_i, \gamma_j] = -\frac{\bar{\gamma}_i\bar{\gamma}_j}{1+k}, \; i \neq j. \tag{70}$$

Proof: Consider only the joint distribution of the ith clone frequency γ_i and the total frequency of other cells $1-\gamma_i$. Using the result from Proposition 5, we know that the joint probability density function for these two frequencies is that of the Beta distribution,

$$P(\gamma_i) = \frac{\Gamma(k)}{\Gamma(k_i)\Gamma(k-k_i)}\gamma_i^{k_i-1}(1-\gamma_i)^{k-k_i-1} \tag{71}$$

which is the marginal distribution of γ_i. The ith clone frequency has the first moment

$$\bar{\gamma}_i = \int_0^1 \gamma_i P(\gamma_i)\, d\gamma_i = \frac{\Gamma(k)}{\Gamma(k_i)\Gamma(k-k_i)}\int_0^1 \gamma_i^{k_i}(1-\gamma_i)^{k-k_i-1} d\gamma_i = \frac{k_i}{k} \tag{72}$$

which is sensible, because the only asymmetry between neutral clones is the asymmetry in seeding rates; hence the average frequency of a clone in a mature metastasis is the fraction of migrants to that metastasis that are in that clone. The second moment is

$$\mathbb{E}\left[\gamma_i^2\right] = \int_0^1 \gamma_i^2 P(\gamma_i)\, d\gamma_i = \frac{\Gamma(k)}{\Gamma(k_i)\Gamma(k-k_i)}\int_0^1 \gamma_i^{k_i+1}(1-\gamma_i)^{k-k_i-1} d\gamma_i = \frac{k_i}{k}\cdot\frac{1+k_i}{1+k} \tag{73}$$

The variance in clone frequency can then be calculated to give

$$\mathrm{Var}[\gamma_i] = \mathbb{E}[\gamma_i^2] - \bar{\gamma}_i^2 = \frac{\frac{k_i}{k}(1-\frac{k_i}{k})}{1+k} = \frac{\bar{\gamma}_i(1-\bar{\gamma}_i)}{1+k}, \tag{74}$$

the desired result. Finally, the crossed second moment for two of the clone frequencies is

$$\mathbb{E}\left[\gamma_i\gamma_j\right] = \frac{\Gamma(k_i)^{-1}\Gamma(k_j)^{-1}\Gamma(k)}{\Gamma(k-k_i-k_j)}\int_0^1\int_0^{1-\gamma_j} \gamma_i^{k_i}\gamma_j^{k_j}(1-\gamma_i)^{k-k_i-k_j-1} d\gamma_i d\gamma_j = \bar{\gamma}_i\bar{\gamma}_j\frac{k}{1+k} \tag{75}$$

for $i \neq j$. The covariance between two clone frequencies is thus

$$\mathrm{Cov}[\gamma_i, \gamma_j] = \mathbb{E}[\gamma_i\gamma_j] - \bar{\gamma}_i\bar{\gamma}_j = -\frac{\bar{\gamma}_i\bar{\gamma}_j}{1+k}, \quad i \neq j. \tag{76}$$

Corollary 5.2. The mean number of clones n in the metastasis that are detected (that is, present at a frequency greater than τ where $0 < \tau < 1$) is given by

$$\bar{n}_{\mathrm{det}} = N - \sum_{i=1}^{N} I_\tau(k_i, k-k_i) \tag{77}$$

where I_x denotes the regularized incomplete beta function.

Proof: The probability that each clone has a frequency less than the detection threshold τ is given by integrating equation 71,

$$P(\gamma_i < \tau) = \frac{\Gamma(k)}{\Gamma(k_i)\Gamma(k-k_i)} \int_0^\tau \gamma_i^{k_i-1}(1-\gamma_i)^{k_i-1} d\gamma_i = I_\tau(k_i, k-k_i)$$

The mean number of clones which are not detected is then

$$N - \bar{n}_{\text{det}} = \sum_{i=1}^{N} I_\tau(k_i, k-k_i)$$

which immediately implies the result to be proven.

Proposition 6. The ratio of the mean clonal diversity of a metastasis $\bar{D}_2$ to the mean clonal diversity of the population of migratory cells in circulation D_1 is

$$\frac{\bar{D}_2}{D_1} = \frac{k}{1+k} \tag{78}$$

Proof: Of the k average migrants per generation, on average k_i are of clone type i, so the probability that any given chosen migratory cell in circulation is of clone type i is k_i/k. The probability that two chosen migratory cells, sampled with replacement, are of the same clonal type is therefore $\sum_{i=1}^{N}(k_i/k)^2$. The mean clonal diversity of this population of migratory cells, defined as the probability that the two sampled cells are different, is then the complement,

$$D_1 = 1 - \sum_{i=1}^{N} \left(\frac{k_i}{k}\right)^2 \tag{79}$$

The mean clonal diversity of the metastasis is instead

$$\bar{D}_2 = 1 - \sum_{i=1}^{N} \mathbb{E}\left[\gamma_i^2\right] = 1 - \sum_{i=1}^{N} \frac{k_i}{k} \cdot \frac{1+k_i}{1+k} \tag{80}$$

where we have used the result of equation 73. Then we see that

$$\bar{D}_2 = 1 - \frac{1}{1+k}\sum_{i=1}^{N} \frac{k_i}{k} - \frac{k}{1+k}\sum_{i=1}^{N}\left(\frac{k_i}{k}\right)^2 \tag{81}$$

Since $k = \sum_{i=1}^{N} k_i$ and $D_1 = 1 - \sum_{i=1}^{N}(k_i/k)^2$, this becomes

$$\bar{D}_2 = 1 - \frac{1}{1+k} - \frac{k}{1+k}(1-D_1) = \frac{k}{1+k}D_1 \tag{82}$$

which immediately implies the result to be proven.

Corollary 6.1. The clonal differentiation index F_{ST} among M metastases is

$$F_{ST} = 1 - \frac{\bar{D}_2}{\bar{D}_2^*} = \Big(1 - \frac{1}{M}\Big)\frac{1}{1+k} \tag{83}$$

where $\bar{D}_2^*$ denotes the mean clonal diversity in the population of cells aggregated across all metastases seeded by the same primary tumor.

Proof: If each metastasis receives k mean migrants per generation, the aggregate population of M metastases receives Mk mean migrants per generation. Otherwise the growth of the aggregate population behaves identically to an individual metastasis, and so by replacing k with Mk in equation 82 we obtain an expression for the clonal diversity across all metastases,

$$\bar{D}_2 = \frac{Mk}{1+Mk} D_1 \tag{84}$$

Using our expressions for $\bar{D}_2$ and $\bar{D}_2^*$, we calculate the clonal differentiation index F_{ST} to be

$$F_{ST} = 1 - \frac{\bar{D}_2}{\bar{D}_2^*} = 1 - \frac{k}{1+k}\frac{1+Mk}{Mk} = \Big(1 - \frac{1}{M}\Big)\frac{1}{1+k} \tag{85}$$

We note that this result does not require all of the metastases to be of equal size, only that their sizes all be much larger than k. We further note that when the number of metastases is large, $M \gg 1$, this implies that $F_{ST} \approx \frac{1}{1+k}$.

Proposition 7. Given the clone frequencies $\bar{\gamma}_i$ of each clone $i = 1, \ldots, N$ in the primary tumor and the analogous clone frequencies γ_{ij} in each metastasis $j = 1, \ldots, M$, the maximum likelihood estimates $\hat{k}_j$ for the total seeding influx k_j to each metastasis j satisfy

$$\sum_{j=1}^{M} \hat{k}_j \cdot \beta_{ij} = 0, \text{ where } \beta_{ij} = \ln \gamma_{ij} - \big[\psi(\bar{\gamma}_i k_j) - \psi(k_j)\big]. \tag{86}$$

Proof: Because the clone frequencies in each metastases are independent when conditioned on the clone seeding rates, the likelihood of the clone frequency data is a simple product over the Dirichlet distribution describing the clone frequencies in each metastasis:

$$P(\gamma) = \prod_{j=1}^{M} \left[\Gamma(k_j) \prod_{i=1}^{N} \frac{\gamma_{ij}^{\bar{\gamma}_i k_j - 1}}{\Gamma(\bar{\gamma}_i k_j)}\right] \tag{87}$$

The associated log-likelihood is

$$\ln P(\gamma) = \sum_{j=1}^{M} \left[\ln \Gamma(k_j) + \sum_{i=1}^{N} \Big[(\bar{\gamma}_i k_j - 1) \ln \gamma_{ij} - \ln \Gamma(\bar{\gamma}_i k_j)\Big]\right] \tag{88}$$

Maximizing the log-likelihood with respect to k_j gives

$$\frac{\partial}{\partial k_j} \ln P(\gamma) = \psi(k_j) + \sum_{i=1}^{N} \Big[\bar{\gamma}_i \ln \gamma_{ij} - \bar{\gamma}_i \psi(\bar{\gamma}_i k_j)\Big] = \sum_{i=1}^{N} \bar{\gamma}_i \cdot \beta_{ij} = 0 \tag{89}$$

Here β_{ij} can be interpreted as the sample bias for the log-scaled clone frequency data, since the expected value of $\ln \gamma_{ij}$ is given by $\psi(\bar{\gamma}_i k_j) - \psi(k_j)$.

Corollary 7.1. The uncertainty in the maximum likelihood estimate $\ln \hat{k}_j$ for the log-scaled seeding influx $\ln k_j$ given the clonal frequencies $\bar{\gamma}_i$ and γ_{ij} is given by

$$\sigma_j^2 = \left[-\hat{k}_j^2 \psi'(\hat{k}_j) + \sum_{i=1}^{N} (\hat{k}_j \bar{\gamma}_i)^2 \psi'(\hat{k}\bar{\gamma}_i) \right]^{-1} \tag{90}$$

such that a 95% confidence interval for k_j can be constructed from the bounds $\hat{k}_j e^{\pm 1.96\sigma_j}$.

Proof: The first derivative of the log-likelihood function 88 with respect to $\ln k_j$ is

$$\frac{\partial \ln P(\gamma)}{\partial \ln k_j} = k_j \frac{\partial \ln P(\gamma)}{\partial k_j} \tag{91}$$

and hence the MLE for $\ln k_j$ is $\ln \hat{k}_j$. The second derivative with respect to $\ln k_j$ is then

$$-\sigma_j^{-2} = \frac{\partial^2 \ln P(\gamma)}{(\partial \ln k)^2} = k_j^2 \frac{\partial^2 \ln P(\gamma)}{\partial^2 k_j} + k_j \frac{\partial \ln P(\gamma)}{\partial k_j} \tag{92}$$

When evaluated at the MLE $k_j = \hat{k}_j$, the final term vanishes, leaving

$$-\sigma_j^{-2} = \hat{k}_j^2 \frac{\partial^2 \ln P(\gamma)}{\partial^2 k_j}\Big|_{\hat{k}} = \hat{k}_j^2 \left[\psi'(\hat{k}_j) - \sum_{i=1}^{N} \bar{\gamma}_i^2 \psi'(\bar{\gamma}_i \hat{k}_j) \right] \tag{93}$$

Inverting both sides gives the desired expression for the uncertainty. For a likelihood function that is approximately log-normal in k_j, we can treat $\ln k_j$ as having a normal distribution with mean $\hat{k}_j$ and variance σ_j^2. Using the z-score of 1.96 for a 95% confidence interval, an upper and lower bound for $\ln k_j$ is then $\ln \hat{k}_j \pm 1.96\sigma_j$, or roughly two standard deviations above and below the mean. Exponentiating gives the desired upper and lower bound for k_j.

Corollary 7.2. Given the clone frequencies $\bar{\gamma}_i$ and γ_i of each clone $i = 1, \ldots, N$ in the primary tumor and a metastasis that it seeded, respectively, and dropping the j subscript, the maximum likelihood estimate $\hat{k}$ for the total seeding influx k follows the scaling law

$$\hat{k} \approx \frac{N-1}{\alpha D_{KL}(\bar{\gamma} \| \gamma)}, \quad \text{where} \;\; D_{KL}(\bar{\gamma} \| \gamma) = \sum_{i=1}^{N} \bar{\gamma}_i \ln \frac{\bar{\gamma}_i}{\gamma_i} \tag{94}$$

where D_{KL} denotes the KL-divergence between the clone frequencies $\bar{\gamma}$ and γ, and α is a constant such that $\alpha = 1$ if $D_{KL}(\bar{\gamma} \| \gamma) \gg N-1$ and $\alpha = 2$ if $D_{KL}(\bar{\gamma} \| \gamma) \ll \frac{N-1}{2} \min_i \bar{\gamma}_i$.

Proof: By proposition 7, we know that the maximum likelihood estimate $\hat{k}$ solves

$$\sum_{i=1}^{N} \bar{\gamma}_i \cdot \Big(\ln \gamma_i - \big[\psi(\bar{\gamma}_i \hat{k}) - \psi(\hat{k}) \big] \Big) = 0 \tag{95}$$

Suppose that $\hat{k} \ll 1$, which implies that $\bar{\gamma}_i \hat{k} \ll 1$ for all i because $\bar{\gamma}_i < 1$. Since the digamma function has the series expansion $\psi(x) = -\frac{1}{x} - c_\gamma + O(x)$, then for $\hat{k} \ll 1$ we find that

$$\sum_{i=1}^{N} \bar{\gamma}_i \cdot \left(\ln \gamma_i + \left[\frac{1}{\bar{\gamma}_i \hat{k}} - \frac{1}{\hat{k}} \right] \right) = \sum_{i=1}^{N} \bar{\gamma}_i \ln \gamma_i + \hat{k}^{-1} \sum_{i=1}^{N} \left(1 - \bar{\gamma}_i\right) \approx 0 \tag{96}$$

Rearranging this equation to solve for $\hat{k}$ gives the approximate scaling law

$$\hat{k} \approx \frac{\sum_{i=1}^{N} \left(1 - \bar{\gamma}_i\right)}{\sum_{i=1}^{N} \bar{\gamma}_i \ln(1/\gamma_i)} = \frac{N-1}{D_{KL}(\bar{\gamma} \| \gamma) + S_1} \tag{97}$$

where D_{KL} is the KL-divergence defined above and $S_1 = \sum_{i=1}^{N} \bar{\gamma}_i \ln(1/\bar{\gamma}_i)$ is the Shannon diversity index of the primary tumor. This approximation is valid only for $\hat{k} \ll 1$, which requires that $D_{KL}(\bar{\gamma} \| \gamma) + S_1 \gg N - 1$. But $S_1 \leq \ln N$, with equality for uniform seeding rates $\bar{\gamma}_i = \frac{1}{N}$. Because $N > \ln N$, we can obtain the validity condition $D_{KL}(\bar{\gamma} \| \gamma) \gg N - 1$, which implies $D_{KL}(\bar{\gamma} \| \gamma) \gg S_1$; neglecting S_1 then gives the desired scaling law with $\alpha = 1$.

At the other extreme, suppose that $\hat{k} \gg 1$, and moreover $\bar{\gamma}_i \hat{k} \gg 1$ for all i, so that we have $\hat{k} \gg 1/\min_i \bar{\gamma}_i$. Using the series expansion $\psi(x) = \ln x - \frac{1}{2x} + O(x^{-2})$, we instead find that

$$\sum_{i=1}^{N} \bar{\gamma}_i \cdot \left(\ln \gamma_i - \left[\ln(\bar{\gamma}_i \hat{k}) - \frac{1}{2\bar{\gamma}_i \hat{k}} - \ln(\hat{k}) + \frac{1}{2\hat{k}} \right] \right) \approx 0 \tag{98}$$

Combining like terms and solving for $\hat{k}$ gives the approximate scaling law

$$\hat{k} \approx \frac{\sum_{i=1}^{N} \left(1 - \bar{\gamma}_i\right)}{2 \sum_{i=1}^{N} \bar{\gamma}_i \ln(\bar{\gamma}_i/\gamma_i)} = \frac{N-1}{2 D_{KL}(\bar{\gamma} \| \gamma)} \tag{99}$$

This is the desired scaling law with $\alpha = 2$, and to ensure the validity condition $\hat{k} \gg 1/\min_i \bar{\gamma}_i$ is satisfied, we require that $D_{KL}(\bar{\gamma} \| \gamma) \ll \frac{N-1}{2} \min_i \bar{\gamma}_i$.

Corollary 7.3. The uncertainty in the maximum likelihood estimate $\ln \hat{k}$ scales as

$$\sigma^2 \approx \frac{\alpha}{N-1} \tag{100}$$

where α is the constant described in Corollary 7.2.

Proof: In the low seeding regime $\hat{k} \ll 1$ considered in Corollary 7.2, we apply the series expansion $\psi'(x) = x^{-2} + O(1)$ to approximate the uncertainty from Corollary 7.1 as

$$\sigma^2 = \left[-\hat{k}^2 \psi'(\hat{k}) + \sum_{i=1}^{N} (\hat{k}\bar{\gamma}_i)^2 \psi'(\hat{k}\bar{\gamma}_i) \right]^{-1} \approx \left[-1 - \sum_{i=1}^{N} 1 \right]^{-1} = \frac{1}{N-1} \tag{101}$$

which is the desired scaling law with $\alpha = 1$. In the high seeding regime $\hat{k} \gg 1/\min_i \bar{\gamma}_i$, we apply the series expansion $\psi'(x) = x^{-1} + \frac{1}{2}x^{-2} + O(x^{-3})$ to instead the approximation

$$\sigma^2 \approx \left[-\hat{k} - \frac{1}{2} + \sum_{i=1}^{N} \left(\hat{k}\bar{\gamma}_i - \frac{1}{2}\right)\right]^{-1} = \left[-\hat{k} + \frac{1}{2} + \hat{k} + \frac{N}{2}\right]^{-1} = \frac{2}{N-1} \tag{102}$$

which is the desired scaling law with $\alpha = 2$. Hence, in both regimes, the behavior of α scales analogously to its behavior shown in Corollary 7.3. With these scaling laws, a 95% confidence interval can be constructed using the upper and lower bounds

$$\hat{k} \cdot e^{\pm 1.96\sigma} = \hat{k} \cdot e^{\pm 1.96\sqrt{\frac{\alpha}{N-1}}} \tag{103}$$

For low seeding with $\alpha = 1$, this can be simplified numerically as $\hat{k} \cdot 7.1^{\pm \frac{1}{\sqrt{N-1}}}$, while for high seeding with $\alpha = 2$, we instead obtain the numerical simplification $\hat{k} \cdot 16^{\pm \frac{1}{\sqrt{N-1}}}$.

Corollary 7.4. Given only the clone frequencies γ_{ij} of each clone $i = 1, \ldots, N$ in each metastasis $j = 1, \ldots, M$, the maximum likelihood estimates for the mean clone seeding frequencies $\hat{\bar{\gamma}}_i$ and the total seeding influx $\hat{k}_j$ to each metastasis jointly satisfy

$$\sum_{i=1}^{N} \hat{\bar{\gamma}}_i \cdot \beta_{ij} = 0 \quad \text{and} \quad \sum_{j=1}^{M} \hat{k}_j \cdot \beta_{ij} = 0 \tag{104}$$

Proof: In order to maximize the log-likelihood from equation 88 subject to the constraint $\sum_{i=1}^{N} \bar{\gamma}_i = 1$, we define the augmented objective function

$$J = \sum_{j=1}^{M} \left[\ln \Gamma(k_j) + \sum_{i=1}^{N} \left[(\bar{\gamma}_i k_j - 1) \ln \gamma_{ij} - \ln \Gamma(\bar{\gamma}_i k_j)\right]\right] - \Lambda \left[1 - \sum_{i=1}^{N} \bar{\gamma}_i\right] \tag{105}$$

where Λ is the Lagrange multiplier. Maximizing this objective J with respect to k_j gives

$$\frac{\partial J}{\partial k_j} = \sum_{i=1}^{N} \bar{\gamma}_i \cdot \left[\ln \gamma_{ij} - \left[\psi(\bar{\gamma}_i k_j) - \psi(k_j)\right]\right] = \sum_{i=1}^{N} \bar{\gamma}_i \cdot \beta_{ij} = 0, \tag{106}$$

precisely the same condition as in Proposition 7. Maximizing with respect to $\bar{\gamma}_i$ gives

$$\frac{\partial J}{\partial \bar{\gamma}_i} = \Lambda + \sum_{j=1}^{M} k_j \cdot \left[\ln \gamma_{ij} - \psi(\bar{\gamma}_i k_j)\right] = 0 \tag{107}$$

Multiplying by $\bar{\gamma}_i$ and summing over all clones $i = 1$ to N gives

$$\sum_{i=1}^{N} \bar{\gamma}_i \frac{\partial J}{\partial \bar{\gamma}_i} = \Lambda + \sum_{j=1}^{M} \left[k_j \sum_{i=1}^{N} \bar{\gamma}_i \cdot \left[\ln \gamma_{ij} - \psi(\bar{\gamma}_i k_j)\right]\right] = 0 \tag{108}$$

Applying condition (106), we can simplify this to obtain

$$\sum_{i=1}^{N} \bar{\gamma}_i \frac{\partial J}{\partial \bar{\gamma}_i} = \Lambda - \sum_{j=1}^{M} \left[k_j \sum_{i=1}^{N} \bar{\gamma}_i \psi(k_j) \right] = \Lambda - \sum_{j=1}^{M} k_j \psi(k_j) = 0 \tag{109}$$

since $\sum_{i=1}^{N} \bar{\gamma}_i = 1$. Hence $\Lambda = \sum_{j=1}^{M} k_j \psi(k_j)$, and so condition (107) becomes

$$\frac{\partial J}{\partial \bar{\gamma}_i} = \sum_{j=1}^{M} k_j \cdot \Big[\ln \gamma_{ij} - \big[\psi(\bar{\gamma}_i k_j) - \psi(k_j) \big] \Big] = \sum_{j=1}^{M} k_j \cdot \beta_{ij} = 0 \tag{110}$$

Therefore the maximum likelihood estimates for k_j and $\bar{\gamma}_i$ are jointly in the nullspace of β_{ij}, such that the sample bias for the log-scaled clone frequency data vanishes when averaged over the metastases proportionally to their influxes k_j, and also vanishes when averaged over the clones proportionally to their seeding frequencies $\bar{\gamma}_i$.

Proposition 8. Consider a tertiary metastasis that is not directly seeded by the primary tumor, but is instead seeded indirectly via a secondary site such as a lymph node or larger metastasis. The ratio of the mean clonal diversity of a mature tertiary tumor D_3 to that of the primary tumor D_1 can be computed as a product over each of the two seeding steps:

$$\frac{D_3}{D_1} = \frac{D_2}{D_1} \times \frac{D_3}{D_2} = \frac{k_1^2}{1 + k_1^2} \times \frac{k_2^3}{1 + k_2^3} \tag{111}$$

where k_1^2 denotes the seeding influx from the primary tumor to the secondary site, and k_2^3 denotes the seeding influx from the secondary site to the tertiary metastasis, assuming that the secondary site reaches a steady size prior to seeding the tertiary site (Fig. S2F, Fig. S7).

Proof: The result is a straightforward application of Proposition 6 independently for each of the two seeding steps, and in principle this approach can be extended for any number of consecutive seeding steps as follows:

$$\frac{D_m}{D_1} = \prod_{j=1}^{m-1} \frac{D_{j+1}}{D_i} = \prod_{j=1}^{m-1} \frac{k_j^{j+1}}{1 + k_j^{j+1}} \tag{112}$$

We also note that the covariance in a clonal frequency γ_i between two tertiary tumors must be equal to the variance in the clonal frequency at the secondary site. By defining an effective flux k_1^3 between the primary and the tertiary sites such that $\frac{D_3}{D_1} = \frac{k_1^3}{1+k_1^3}$, we find that this effective flux is given by the harmonic mean of the stepwise fluxes k_1^2, k_2^3 and their product,

$$k_1^3 = \frac{k_1^2 k_2^3}{1 + k_1^2 + k_2^3} = \left(\frac{1}{k_1^2} + \frac{1}{k_2^3} + \frac{1}{k_1^2 k_2^3} \right)^{-1} \tag{113}$$

It follows that, when only one seeding step is limiting and the other is very rapid, then k_1^3 is approximately equal to the slower of the two stepwise influxes k_1^2 and k_2^3, such that we have $k_1^3 \approx \min\{k_1^2, k_2^3\}$, while if both steps are very slow, then k_1^3 is approximately given by the product of the two stepwise fluxes, $k_1^3 \approx k_1^2 \times k_2^3$, as is typical of multistep reaction dynamics.

Corollary 8.1. In the case where every pair of tumors has the same seeding influx, $k_j^{j+1} = k$, the expected time until the secondary site seeds a surviving lineage in a tertiary tumor (or equivalently reseeds a surviving lineage in the primary tumor) is given by

$$\bar{T}^* = r^{-1}\Big(\ln \frac{\rho Y}{k^2} - c_\gamma \Big) \tag{114}$$

for realistically large tumor sizes $Y \gg \frac{k^2}{\rho}$, where $c_\gamma \approx 0.577$ is the Euler-Mascheroni constant.

Proof: The expected number of surviving lineages in the tertiary tumor $\bar{L}_3$ is the product of the mean size of the secondary tumor $\bar{y}(t)$ that seeds it (derived in Proposition 1), the per-cell seeding rate $\ell = \lambda/Y$, and the lineage survival probability ρ, integrated over time:

$$\bar{L}_3(t) = \frac{\rho\lambda}{Y} \int_0^t \bar{y}(\tau) d\tau = \frac{k^2}{\rho Y}\big(e^{rt} - 1 - rt\big) \tag{115}$$

In terms of the factor $\epsilon = \frac{k^2}{\rho Y}$, the time T^* at which the first cell from the secondary site seeds a surviving lineage in another site follows a time-inhomogeneous exponential distribution,

$$f_T^*(t) = \dot{\bar{L}}_3(t) e^{-\bar{L}_3(t)} = \epsilon r(e^{rt} - 1) \exp\big\{ -\epsilon(e^{rt} - 1 - rt)\big\} \tag{116}$$

where ϵ is exceedingly small since $Y \gg \frac{k^2}{\rho}$. The mean value of T^* is then obtained as

$$\bar{T}^* = \int_0^\infty t f_T^*(t) dt = \int_0^\infty \epsilon r t(e^{rt} - 1) \exp\big\{ -\epsilon(e^{rt} - 1 - rt)\big\} dt. \tag{117}$$

Using the change of variables $u = \epsilon e^{rt}$, we can rewrite this integral in terms of the incomplete gamma function $\Gamma_\alpha(z) = \int_\alpha^\infty u^{z-1} e^{-u} du$ as follows:

$$r\bar{T}^* = \Big(\frac{e}{\epsilon}\Big)^\epsilon \int_\epsilon^\infty u^{\epsilon-1} e^{-u} du = \Big(\frac{e}{\epsilon}\Big)^\epsilon \Gamma_\epsilon(\epsilon). \tag{118}$$

Since $\left(\frac{e}{\epsilon}\right)^\epsilon \approx 1$ for small $\epsilon \ll 1$, then $r\bar{T}^* \approx \Gamma_\epsilon(\epsilon)$. Either expression has the series expansion

$$r\bar{T}^* = -\ln\epsilon - c_\gamma + O(\epsilon) \tag{119}$$

for small ϵ, and dividing through by the growth rate r gives precisely the result to be proved.

Corollary 8.2. At the expected time $\bar{T}^*$ at which the secondary tumor first seeds a surviving lineage in another tumor, the expected size of the secondary tumor is a fraction $56\%/k$ of its mature size Y.

Proof: The expected size of the secondary tumor at any time t is given by Proposition 1, and $\bar{T}^*$ is given by Corollary 8.1. Substituting the latter into the former gives

$$\frac{\bar{y}(\bar{T}^*)}{Y} = \frac{k}{\rho Y}\Big(e^{r\bar{T}^*} - 1\Big) \approx \frac{k}{\rho Y} e^{r\bar{T}^*} = \frac{e^{-c_\gamma}}{k} \tag{120}$$

where the approximation holds for realistically large tumors $Y \gg \frac{k^2}{\rho} e^{1+c_\gamma}$. Because we know that $e^{-c_\gamma} \approx 0.5615$, we conclude that the ratio of the expected secondary tumor size at the time it first seeds a surviving lineage to its mature size Y is given by $56.15\%/k$. For a seeding rate in the range $1 < k < 10$, this fraction would be in the range 5.615% to 56.15%. With exponential tumor growth, both of these values are near the end of a tumor's growth phase.

Corollary 8.3. At the most likely time $\hat{T}$ at which the secondary tumor is detected, the expected number of surviving lineages that it will have seeded at some other site (such as a different metastasis or the primary tumor) is nearly equal to the seeding influx k.

Proof: The mean number of seeded surviving lineages is given by equation 115 to be

$$\bar{L}_3(\hat{T}) = \epsilon\Big(e^{r\hat{T}} - 1 - r\hat{T}\Big) \tag{121}$$

with $\epsilon = \frac{k^2}{\rho Y}$ as before. Substituting in the median detection time $\hat{T}$ from Proposition 3,

$$\bar{L}_3(\hat{T}) = \epsilon\Big(\frac{\rho Y}{k} - 1 - \ln\frac{\rho Y}{k}\Big) = k - \epsilon\Big(1 + \ln k - \ln\epsilon\Big) \approx k \tag{122}$$

where we can justify the approximation because $\epsilon \ln k \ll 1$ and $|\epsilon \ln \epsilon| \ll 1$ for small $\epsilon \ll 1$. Therefore the seeding influx k is not only the expected number of cells transferred from the primary tumor to the secondary tumor per generation, but also the expected number of cells transferred from the secondary tumor to another site during the entire tumor growth phase.

References

[1] Andrew McPherson, Andrew Roth, Emma Laks, Tehmina Masud, Ali Bashashati, Allen W. Zhang, Gavin Ha, Justina Biele, Damian Yap, Adrian Wan, Leah M. Prentice, Jaswinder Khattra, Maia A. Smith, Cydney B. Nielsen, Sarah C. Mullaly, Steve Kalloger, Anthony Karnezis, Karey Shumansky, Celia Siu, Jamie Rosner, Hector Li Chan, Julie Ho, Nataliya Melnyk, Janine Senz, Winnie Yang, Richard Moore, Andrew J. Mungall, Marco A. Marra, Alexandre Bouchard-Côté, C. Blake Gilks, David G. Huntsman, Jessica N. McAlpine, Samuel Aparicio, and Sohrab P. Shah. Divergent modes of clonal spread and intraperitoneal mixing in high-grade serous ovarian cancer. *Nature Genetics*, 48(7):758–767, 2016.

[2] Peter Savas, Zhi Ling Teo, Christophe Lefevre, Christoffer Flensburg, Franco Caramia, Kathryn Alsop, Mariam Mansour, Prudence A. Francis, Heather A. Thorne, Maria Joao Silva, Nnennaya Kanu, Michelle Dietzen, Andrew Rowan, Maik Kschischo, Stephen Fox, David D. Bowtell, Sarah Jane Dawson, Terence P. Speed, Charles Swanton, and Sherene Loi. The Subclonal Architecture of Metastatic Breast Cancer: Results from a Prospective Community-Based Rapid Autopsy Program "CASCADE". *PLoS Medicine*, 13(12), 2016.

[3] Richard Durrett. Branching process models of cancer. In *Branching Process Models of Cancer*, pages 1–63. 2015.

[4] J.G. Reiter, A.P. Makohon-Moore, J.M. Gerold, A. Heyde, M.A. Attiyeh, Z.A. Kohutek, C.J. Tokheim, A. Brown, R.M. DeBlasio, J. Niyazov, and A. Zucker. Minimal functional driver gene heterogeneity among untreated metastases. *Science*, 361(6406):1033–1037, 2018.

[5] Philipp M Altrock, Lin L Liu, and Franziska Michor. The mathematics of cancer: Integrating quantitative models. *Nature Reviews Cancer*, 15(12):730–745, 2015.

[6] Niko Beerenwinkel, Roland F. Schwarz, Moritz Gerstung, and Florian Markowetz. Cancer evolution: Mathematical models and computational inference. *Systematic Biology*, 64(1):e1–e25, 2015.

[7] D Wodarz and N L Komarova. *Computational Biology of Cancer: Lecture Notes and Mathematical Modeling.* World Scientific Pub Co Inc, 2005.

[8] K B Athreya and P E Ney. *Branching Processes.* Springer-Verlag Berlin Heidelberg, 1972.

[9] Marek Kimmel and David E Axelrod. *Branching Processes in Biology*, volume 19. Springer-Verlag New York, 2002.

[10] Marc J. Williams, Benjamin Werner, Chris P. Barnes, Trevor A. Graham, and Andrea Sottoriva. Identification of neutral tumor evolution across cancer types. *Nature Genetics*, 48(3):238–244, 2016.

[11] Luis A. Diaz, Richard T. Williams, Jian Wu, Isaac Kinde, J. Randolph Hecht, Jordan Berlin, Benjamin Allen, Ivana Bozic, Johannes G. Reiter, Martin A. Nowak, Kenneth W. Kinzler, Kelly S. Oliner, and Bert Vogelstein. The molecular evolution of acquired resistance to targeted EGFR blockade in colorectal cancers. *Nature*, 486(7404):537–540, 2012.

[12] Ivana Bozic and Martin A. Nowak. Timing and heterogeneity of mutations associated with drug resistance in metastatic cancers. *Proceedings of the National Academy of Sciences*, 111(45):15964–15968, 2014.

[13] Ivana Bozic, Jeffrey M. Gerold, and Martin A. Nowak. Quantifying clonal and subclonal passenger mutations in cancer evolution. *PLoS Computational Biology*, 12(2), 2016.
